# Immune mechanisms mediating the heterologous effects of BCG vaccination: a systematic review

**DOI:** 10.3389/fimmu.2025.1567111

**Published:** 2025-05-19

**Authors:** Louis Torracinta, Nino Gogichadze, Rachel Tanner

**Affiliations:** ^1^ Medical Sciences Division, University of Oxford, Oxford, United Kingdom; ^2^ Experimental Tuberculosis Unit, Microbiology Department, Germans Trias i Pujol Research Institute (IGTP) and Hospital (HUGTIP), Badalona, Spain; ^3^ Department of Biology, University of Oxford, Oxford, United Kingdom

**Keywords:** BCG, heterologous effects of vaccination, trained immunity, T cells, humoral immunity, tuberculosis

## Abstract

**Introduction:**

BCG vaccination can have heterologous or non-specific effects (NSE) that confer resistance against pathogens other than its target Mycobacterium tuberculosis, but the underlying mechanisms are not fully understood.

**Methods:**

We conducted a systematic review synthesising existing literature on immune mechanisms mediating the heterologous/NSE of BCG. Searches were conducted using MEDLINE and Scopus.

**Results:**

1032 original records were identified, of which 67 were deemed eligible. Several potentially relevant immune pathways were identified, although there may be variation by pathogen. Recent studies have focused on trained immunity whereby innate cells, or the hematopoietic stem and progenitor cells from which they are derived, undergo epigenetic and metabolic reprogramming allowing them to respond more effectively to antigen exposures unrelated to the original stimulus. However, other processes such as granulopoiesis and cross-reactive adaptive immunity may also play a role. Heterologous immunity and NSEs may be influenced by several endogenous and exogenous variables.

**Discussion:**

We discuss the quality of available data, the importance of understanding mechanisms of heterologous protection, and its implications for vaccination strategies.

**Systematic review registration:**

https://www.crd.york.ac.uk/PROSPERO/view/CRD42023400375, identifier CRD42023400375.

## 1 Introduction

The Bacillus Calmette–Guérin (BCG) vaccine has been the only licensed vaccine for tuberculosis (TB) since 1921 (1). It is the most widely used vaccine to date, has a well-established safety profile, and confers protection against severe forms of TB and associated mortality in infants (2), although efficacy against adult pulmonary TB is highly variable. Mounting evidence suggests that BCG may also confer protection against non-mycobacterial infections (3). Calmette, co-developer of BCG, first noted a four-fold reduction in infant mortality in preliminary studies, surpassing expectations if protection were against TB alone. The British government observed a similar phenomenon following the universal introduction of BCG in 1953 (4, 5). Subsequent epidemiological and observational studies have supported heterologous benefits across different populations; a World Health Organisation (WHO) review of 17 birth cohorts found that BCG reduces infant mortality by five to ten deaths per thousand children in the first three years of life (6). This trend has been replicated across different study designs, including systematic reviews of randomised control trials (RCTs) alone (7) or cohort, case-control studies, and RCTs together (8), as well as recent gold-standard blinded RCTs (9, 10) reporting beneficial effects against heterologous infections during the neonatal period in particular.

BCG immunotherapy has been used to treat non-invasive bladder cancer for decades, though the mechanisms remain unclear (11). Additionally, a growing body of literature has suggested that BCG could confer protective benefits against a range of maladies running the gamut from infectious disease (e.g., malaria (12), influenza (13) and HIV-1 (14)); autoimmune disease (e.g., asthma (15) and type 1 diabetes (16)); as well as diseases of later life (e.g., lung cancer (17) and Alzheimer’s (18)), although much evidence remains to be validated. The recent COVID-19 pandemic sparked interest in the potential of BCG to protect against COVID-19 until specific vaccines were available. A large RCT found that BCG did not reduce risk of COVID-19, but the pandemic underscored the need to better understand heterologous immunity (19).

While the terms ‘heterologous effects’ and ‘non-specific effects’ are often used interchangeably, the latter implies the exclusion of antigen cross-reactivity and we will henceforth use ‘heterologous effects’ to encompass both antigen-independent NSE and potential cross-reactivity by cells of the adaptive arm of the immune system. The immune mechanisms mediating the heterologous effects of BCG have been under investigation for over 60 years (20). Studies on macrophage activation using BCG stimuli from the 1960s supported the idea that BCG induces non-specific antibacterial immunity (21, 22). Recently, BCG has been key to unravelling the concept of ‘trained immunity’, whereby the innate immune system retains memory via long-term reprogramming. Upregulation of innate cells and their functional mechanisms by BCG can enhance non-specific protection against unrelated secondary infections (23).

In summary, the idea that BCG vaccination confers heterologous protection against diseases other than TB – despite some uncertainties (24, 25) – is now widely accepted, with many studies exploring the mechanism(s) by which such protection occurs. A better understanding of such heterologous effects could support the development of low-cost interventions to reduce all-cause mortality in infants and could be central to rapid responses against new emerging pathogens during the lag-phase of vaccine development (26). Therefore, we conducted the first systematic review of literature describing the immune mechanism(s) mediating the heterologous effects of BCG.

## 2 Methods

A systematic review of the literature on immune mechanisms mediating the heterologous effects of BCG vaccination was performed in accordance with the Preferred Reporting Items for Systematic reviews and Meta-Analyses (PRISMA) (**Supplementary Table 1**) (27). The study protocol was registered with PROSPERO prior to commencement on 13^th^ March 2023 (ID CRD42023400375). Amendments to the initial protocol were published on 11^th^ June 2024 and 7^th^ April 2025 (28).

### 2.1 Data sources and search strategy

Searches were conducted in the MEDLINE database via PubMed and through Scopus on 6^th^ March 2023. Relevant literature reviews were also screened for additional references. References were then compiled, de-duplicated, and screened using Rayyan, an online web tool for systematic reviews (29). The full search strategy and terms are provided in **Supplementary Table 2**. Eligible studies were imported into EndNote 20 for further review. To ensure the review was not outdated at time of submission, a further search was performed in PubMed using the same strategy and criteria but with a date filter of records published between 6^th^ March 2023 and date of second search (26^th^ September 2024). As this was not part of the core systematic review, additional papers identified are included in the discussion section.

### 2.2 Study selection and eligibility criteria

Studies were considered eligible if they evaluated the potential immune mechanisms of heterologous effects of BCG vaccination in protecting against infectious diseases other than TB in i) persons, cohorts or populations immunised with BCG (or biological samples thereof), ii) preclinical models whereby animals were immunised with BCG (or biological samples thereof), or iii) relevant *in vitro* models. Eligibility also required exposure to, or stimulation with, heterologous pathogens or their antigens *in vivo* or *in vitro*. Any experimental design, formulation, strain, dose and route of administration of the BCG vaccine was accepted.

Studies that described or observed the heterologous effects of BCG vaccination but did not evaluate the potential underlying immune mechanisms, or did so only *in silico*, were excluded. This review does not include studies that solely examine the role of BCG as an immunotherapy for bladder cancer or other tumours; the effect of BCG vaccination on non-infectious disease; the impact of BCG vaccination on other immunisations; or the Mantoux/Tuberculin test. Other exclusion criteria were i) studies in languages other than English, ii) pre-prints or studies that were otherwise not peer-reviewed, iii) studies for which abstracts or full papers were not available, and iv) background articles or reviews containing no original data. Records were screened by at least two authors to determine eligibility. In case of disagreement, a third author screened the record and a consensus was reached.

### 2.3 Data extraction

Data was collected manually from eligible papers by both NG and LT and reviewed by LT. Data was collected, where available and according to relevance, on: i) study groups analysed (species, geographical region, cohorts, age, sample size, interventions); ii) BCG vaccination strain, dose and route of administration; iii) outcomes associated with heterologous protection (or surrogates thereof); iv) immune parameters measured; and v) any associations between immune parameters measured and heterologous protection (or surrogates thereof).

### 2.4 Risk of bias and quality assurance

Risk of meta-biases was reduced by predefining the eligibility criteria, using broad and inclusive search terms, multiple databases, and with no restriction on date of eligibility (up to date of search). To evaluate the quality of the included studies, a quality assurance framework was applied based on previous tools developed by Tanner et al. (30), the ‘Quality Assessment of Controlled Intervention Studies’ tool developed by the National Heart, Lung and Blood Institute (NHLBI) and the ‘Quality Assessment Tool for *in vitro* Studies’ (QUIN) (31). Articles were assigned scores ranging from 0 to 12, classified into the following categories: 0-2 (poor), 3-5 (fair), 6-8 (good), 9-10 (very good), and 11-12 (excellent).

## 3 Results

### 3.1 Identification of studies and their characteristics

**Figure 1:**
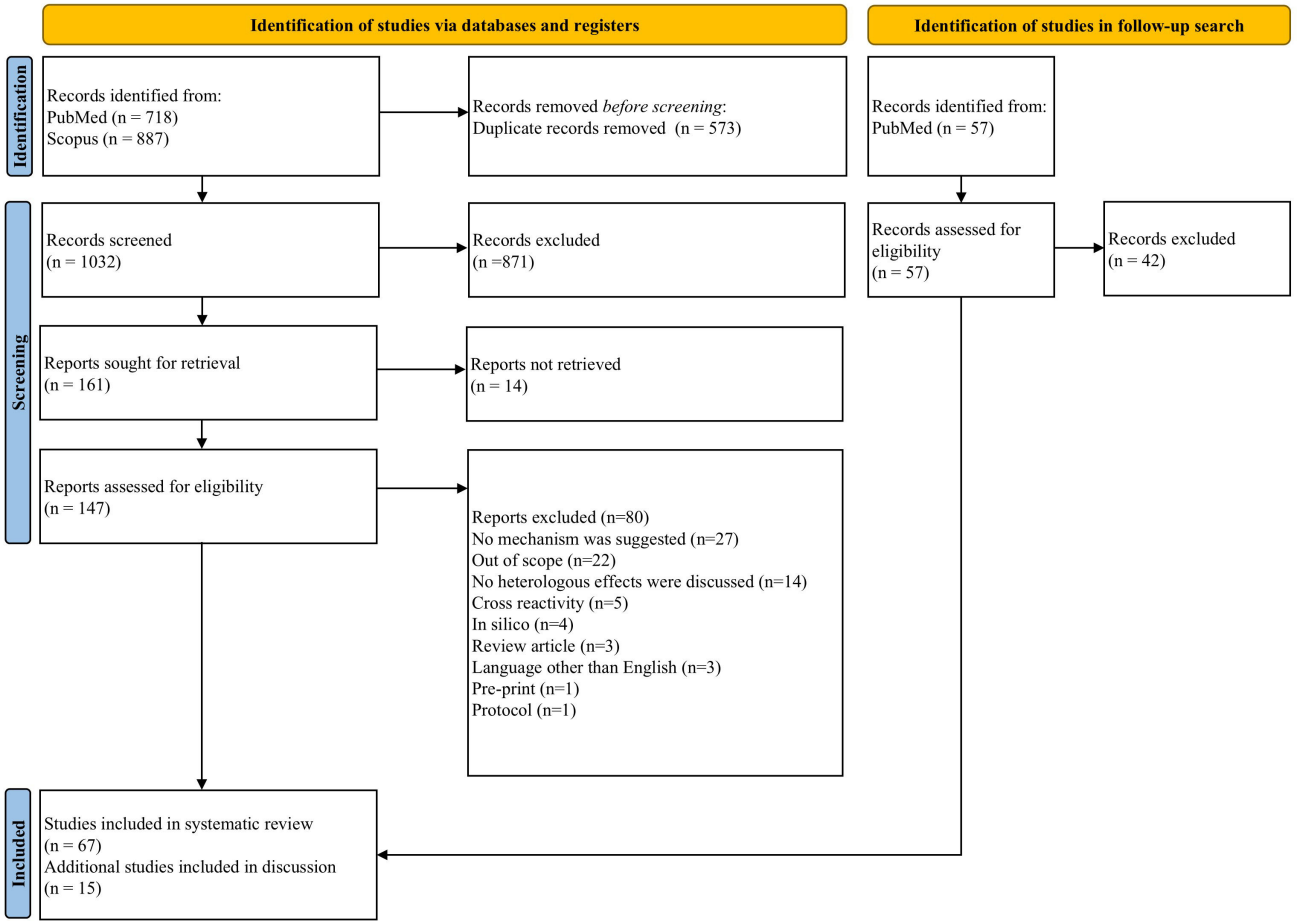
PRISMA (Preferred Reporting Items for Systematic reviews and Meta-Analyses) flow diagram of search process and publication selection.

Searches identified 1032 unique records, of which 161 were sought for full-text screening. Of these, 147 studies were retrievable and were full text screened, while 14 studies could not be retrieved. 67 articles were deemed eligible and included in the review (**Figure 1**) (27).

Of the 80 records excluded after full-text review, 27 were excluded because they did not consider immune mechanism(s) underlying heterologous effects. 22 articles were deemed out of the scope of this review. 14 articles did not discuss heterologous effects in detail. Five articles were related to cross-reactivity of vaccines. Four *in silico* papers were excluded, as were three review articles. Three non-English papers, one pre-print and an experimental protocol were also excluded.

Data extracted from eligible papers is detailed in **Supplementary Table 3**. 38 used human volunteers, 26 were animal studies and three used both human and animal samples. In our independent quality assessment (QA) of the included research, 11 studies (~16%) were deemed ‘excellent’, the majority of which (seven) were concerned with causes of variation in the heterologous effects of BCG vaccination. Two ‘excellent’ studies were concerned with trained innate immunity, and one each with neutrophils and T cells. 14 studies (~21%) were deemed ‘very good’, 38 (~57%) were ‘good’, and four (~6%) were of ‘fair’ quality. Each of these categories was proportionately spread across the different mechanistic categories. None of the included studies were deemed ‘poor’ (**Supplementary Table 4**, **Supplementary Figure 1**).

In the follow-up search, 57 new records were returned of which 15 were deemed eligible.

### 3.2 Immune mechanisms mediating the heterologous effects of BCG vaccination

**Figure 2:**
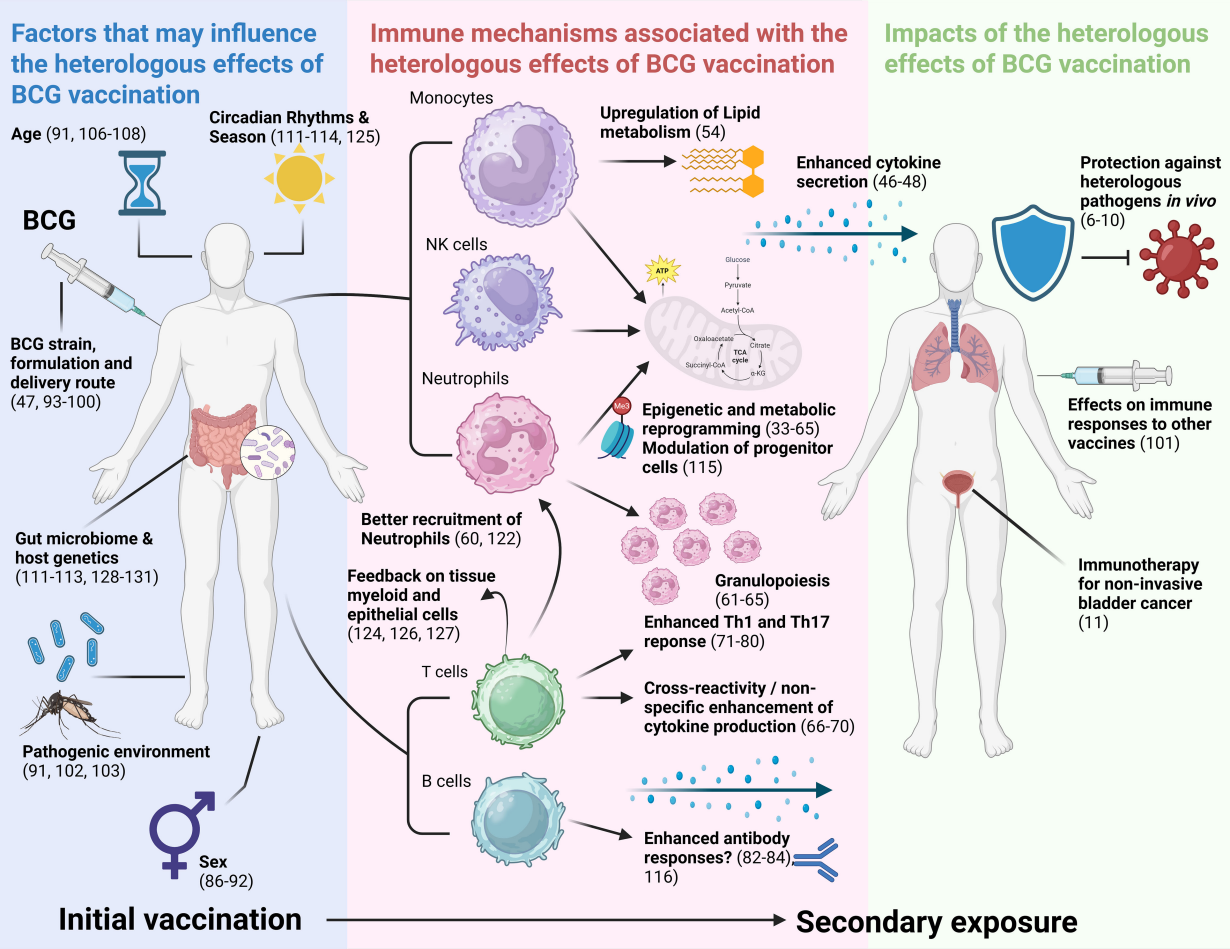
Visual summary of results of this review. Results are synthesised into factors that may influence the NSE of BCG vaccination, immune mechanisms associated with the NSE of BCG vaccination, and impacts of the NSE of BCG vaccination. NSE, non-specific effects; Th1, T helper 1; Th17, T helper 17. Created in BioRender.

Our review suggests that BCG vaccination likely confers heterologous immunity through various mechanisms that differ by context and population, and that its heterologous effects are potentially influenced by several external factors (**Figure 2**). This review will consider each pathway in turn.

#### 3.2.1 Innate Immunity

##### 3.2.1.1 Trained immunity

The concept of trained immunity – that innate cells could retain memory conferring an improved response against secondary exposures – had long been demonstrated in other species (32). This is distinct from the well-understood adaptive immune memory in which antigen-specific memory T and B cells are formed allowing for a faster and more efficient immune response upon subsequent exposure to the same pathogen. Instead, in trained immunity, innate cells undergo long-lasting changes that make them more responsive to subsequent, often unrelated, exposures.

###### 3.2.1.1.1 Monocytes and macrophages

In 2012, Kleinnijenhuis et al. first comprehensively described this phenomenon in humans post-BCG vaccination, though post-BCG non-specific macrophage activation has been reported since 1961 (33–39). In the Kleinnijenhuis study, monocytes collected from Dutch adults vaccinated with BCG produced more cytokines (particularly IFN-γ, IL-1β, and TNF-α) in response to secondary *in vitro* exposure to unrelated pathogens such as *Staphylococcus aureus*, as well as to *Mycobacterium tuberculosis* (*M.tb*) as expected. This heterologous response was shown to be dependent on functional NOD2 proteins as well as epigenetic H3K4me3 methylation. Transcriptomic analyses of gene polymorphisms involved in autophagy also revealed that H3K4 trimethylation was significantly increased in individuals bearing the *ATG2B* autophagy gene (40–43). Other cytokines produced by monocytes such as IL-32 have also been implicated, particularly in the heterologous effects against parasitic infections (44). Further *in vitro* studies have demonstrated that similar trained immunity processes occur in human cord blood adherent monocytes, which are more representative of the monocyte processes of neonatal blood *in vivo* (45).

It has been shown that β-glucan stimuli drive epigenetic modifications for trained immunity through complex metabolic changes in cells (49). Studies from the 1970s noted increased metabolic rates and glycolysis in activated macrophages (50). However, the detailed metabolic effects of BCG vaccination were not reported until 2016, showing that BCG ligands detected by pattern recognition receptors (PRRs) initiate cascades in monocytes/macrophages that epigenetically upregulate glycolysis, glutamine, and glutathione metabolism, along with oxidative phosphorylation, likely via the Akt/mTOR pathway (51–53). If glycolysis or glutaminolysis is inhibited (e.g., by metformin, an mTOR prohibitor), epigenetic changes in H3K4me3 promoter sites are reversed. This is because methylation is regulated by lysine demethylases and histone methyltransferases, whose activity are influenced by metabolites functioning as co-factors. These findings point to an internal feedback loop within the epigenetic and metabolic mechanisms of trained immunity (49). A separate study of neonatal metabolomes found that BCG vaccination alters plasma lipid metabolism and these changes are correlated with blood cytokine responses to later stimulation with multiple Toll-like receptor (TLR) agonists (54). This study, the first of its kind incorporating comprehensive metabolomics in a resource-limited setting, presents a promising new lipid metabolic mechanism that may mediate heterologous effects in early life.

Finally, although hepcidin-mediated hypoferremia, effectively a limit on iron availability for pathogens, has been hypothesised as a metabolic pathway of trained immunity, studies have been unable to demonstrate this effect following BCG vaccination (9, 55). A lesser-known study of protein and zinc-deficient guinea pigs found that BCG vaccination still conferred heterologous effects (56), indicating that these metabolites may not play a mechanistic role, and lipid or glucose-based pathways seem most pertinent given their circulating levels are known predictors of trained immunity in humans (57). Other studies have investigated the role of nitric oxide production in trained immunity, but found no effect (58).

###### 3.2.1.1.2 Natural killer cells

Experiments in natural killer (NK) cells have also demonstrated that BCG vaccination leads to trained immunity and increased secretion of the proinflammatory cytokines IL-1β, TNF-α, and IFN-γ following heterologous stimulation, indicating that this phenomenon occurs across several innate cell types (46–48).

###### 3.2.1.1.3 Neutrophils

Early studies suggested BCG vaccination confers heterologous immunity even in granulocytopenic mouse models, suggesting a limited role for neutrophils in this phenomenon (59). However, a 1990s study found that BCG vaccination in mice enhanced the ability of macrophages and T cells to recruit neutrophils to infection sites weeks later (60), and granulopoiesis is induced through G-CSF stimulation (61). BCG vaccination is now thought to prevent neonatal sepsis mechanistically through rapid granulopoiesis (62). A study analysing hematopoietic stem cell transcriptomes of healthy human volunteers inoculated with BCG found that the genes that are most differentially expressed at three months post-vaccination are those implicated in neutrophil activation and degranulation pathways, ultimately skewing the myeloid lineage toward granulopoiesis. This may drive a phenotypic change marked by increased activation markers and reduced immunosuppression markers (63). Further studies have found BCG stimulation increases production of the antimicrobial chemokine IL-8 and the serine protease elastase in neutrophils upon *ex vivo* exposure to secondary pathogenic stimuli (64). In summary, BCG boosts neutrophil production, activates resting cells, and enhances their antimicrobial function.

As discussed, trained immunity in other innate cells occurs through complex metabolic and epigenetic changes (45). Similarly, neutrophil activation in this context likely involves these pathways. Neutrophils primarily use glycolysis for energy, and *ex vivo*-stimulated neutrophils show increased glycolytic rates following BCG vaccination, along with elevated H3K4me3 modifications at promoters of glycolysis-regulating genes such as phosphofructokinase and mTOR (64). Neutrophils only survive for a few days, yet both the epidemiological and laboratory-based epigenetic data point to months-long heterologous effects, meaning some of these modifications must be to neutrophil precursors, a process supported by current laboratory data on neutrophil activation using β-glucan (65). Further *in vitro* work similar to the metabolomics studies recently attempted in peripheral blood mononuclear cells (PBMCs) (43, 57) measuring epigenetic modifications could therefore be of interest in the context of neutrophils.

#### 3.2.2 Adaptive immunity

##### 3.2.2.1 T cells

BCG may also confer longer-term heterologous effects through adaptive immune mechanisms. Given the degenerative nature of T cell recognition, the conformational shifts a T cell receptor (TCR) undergoes to recognise a peptide/major histocompatibility complex (MHC) complex, and the conserved nature of many microbial antigens, specific memory T cells may in fact be activated by unrelated pathogens through cross-reactivity (66). However, such a phenomenon fails to explain the increased responses to pathogens such as *Candida albicans* or *Staphylococcus aureus* observed following BCG vaccination despite a lack of shared or similar epitopes. Alternatively, BCG-induced proinflammatory cytokine release by lymphocytes may act non-specifically to activate bystander macrophages resulting in a state of temporarily heightened innate immunity, although this effect wanes rapidly. Effector and memory CD8+ T cells can be non-specifically activated by IL-12 and IL-18 during early secondary infection, independent of TCR signalling, leading to IFN-γ secretion that affects innate cells. This provides a plausible explanation for longer-lived T cell-mediated heterologous effects (67).

In 1984, Orme et al. noted the emergence of a splenic T cell population that was temporally associated with the development of an acquired capacity for non-specific resistance to secondary facultative intracellular bacterial pathogens including *Listeria monocytogenes* following intravenous (IV) inoculation of mice with high-dose BCG (68). Further studies suggested BCG-related non-specific resistance to Listeria was lower in T cell-deficient than in intact mice (69, 70). Furthermore, Mathurin et al. found that BCG vaccination rendered mice partially resistant to infection with vaccinia virus; an effect which was lost after CD4+ T cell depletion or inhibition of TCR signalling (71). Nonetheless, the anti-malarial protection from BCG vaccination diminishes after CD8+, but not CD4+, T cell depletion (72). Although non-specifically activated, certain immune mechanisms may be more relevant for some infections than others.

Interestingly, in mice vaccinated with BCG, protection against experimental cerebral malaria after *Plasmodium berghei* infection has been linked to lower levels of proinflammatory mediators in the brain compared to mice infected without BCG vaccination (73). BCG vaccination was also associated with altered T cell phenotypes in the blood and spleen, and a reduced influx of T cells into the brain, which can otherwise have major pathogenic roles. The authors concluded that preventing experimental cerebral malaria results from the anti-inflammatory and T cell inhibitory functions of BCG, rather than from the hypothesised reduction in parasitaemia due to trained innate immunity (73). Such immunosuppressive functions were described over 40 years ago, including early studies describing non-specific T cell suppressor activity following IV inoculation of mice with high-dose BCG (74, 75).

Delaying BCG vaccination to 8 weeks of age does not affect overall T cell proliferation or cytokine polyfunctionality. However, infants vaccinated at birth show a significantly higher frequency of IL-2+ CD8+ T cells in response to *Bordetella pertussis* compared to unvaccinated infants (76). A larger multi-site study comparing BCG strains noted that infants vaccinated with BCG-Denmark mounted significantly higher magnitudes and polyfunctionality of CD4+ T cell responses to *in vitro* stimulation with Tetanus toxoid and *B. Pertussis* antigens compared to those vaccinated with BCG-Bulgaria or BCG-Russia (77). In healthy healthcare workers exposed to SARS-CoV-2, BCG vaccination was associated with enhanced central and effector memory CD4+ and CD8+ T cell subsets overall as long as 3 months later (78), although a study of Danish infants found minimal subset differences (78, 79). BCG may also enhance heterologous Th1 and Th17 immune responses for as long as 1 year after vaccination (80). Non-specific cellular cytotoxicity has been poorly studied, but an early paper measuring cytotoxicity against a non-specific target noted marked changes in NK cell, and to a lesser extent, T cell cytotoxicity following BCG vaccination (81).

##### 3.2.2.2 B cells

Memory B cells are prone to activation by polyclonal stimulation, and it has long been suggested that mycobacterial antigens or T cell cytokines could activate pathogen-specific memory B cells in a non-specific manner, leading to expansion of antibody-secreting cells (82). Early work noted that sera from BCG vaccinated rabbits contained antibodies that bound to radiolabelled antigens from both unrelated and homologous test antigens (83). A recent study by Kimuda et al. found that active TB disease was linked to higher titres of antibodies specific to RSV and measles virus, but BCG vaccination did not affect antibodies against heterologous pathogens in the same way (84). Additional research is needed to clarify how adaptive immunity contributes to the heterologous effects of BCG and the associated mechanisms.

### 3.3 Causes of variation

Some of the studies in this systematic review are large RCTs characterising heterologous effects of BCG vaccination *in vivo* (9, 55, 85–88). The largest trial in Ugandan infants prospectively measured all-cause infectious morbidity, providing compelling evidence for heterologous effects (9). However, RCTs paint a muddled picture of the immune parameters that could mechanistically explain this phenomenon. In Uganda, BCG was associated with some minor epigenetic changes in PBMCs, but significant effects on cytokine production were not observed following stimulation with *Escherichia coli* and *Candida albicans* (9). Similarly, stimulation of cells with *E. coli* and *C. albicans* did not increase rates of cytokine secretion in Dutch infants (85). However, a retrospective study of low birth weight infants in Guinea-Bissau found that both very low and very high cytokine responses to Lipopolysaccharide (LPS) and Phytohemagglutinin (PHA) stimuli were associated with high mortality, and a balanced production was preferable (86). Two Australian studies found only decreased IFN-γ and IL-6 following stimulation with heterologous antigens (87, 88).

Given that heterologous effects *in vitro* are reproducible, what is causing these observed variations? What are the difficulties inherent to eliciting mechanistic trends *in vivo*? This review identified several factors that may explain the population-level variation observed in the heterologous effects of BCG.

#### 3.3.1 Sex differences

There is some evidence suggesting that heterologous vaccine responses can be sex-differential (89). Two Australian infant BCG trials found a significant interaction between sex and macrophage migration inhibitory factor, as well as other cytokine secretion, following heterologous stimulation. Meanwhile, two trials in Guinea-Bissau found smaller, but still notable, variation in cytokine profiles in response to TLR agonists between sexes (86–88, 90). A separate trial measuring BCG and hepatitis B co-vaccination in neonates reported that males produced more IFN-γ and TNF-α, and less MCP-1 in response to heterologous pathogens compared with females (91). Finally, a trial of 307 healthy adults found that BCG down-regulates systemic inflammation alongside enhanced cytokine responses, but mainly in male cohorts, likely due to circulating testosterone levels (92). The mechanisms underlying the sex differential effects in heterologous immunity are largely unknown, and small demographic imbalances in study populations may drive observed variation.

#### 3.3.2 Vaccine strain and method of delivery

A number of studies suggest that the BCG strain affects heterologous effects; specifically, slow growth and live batches elicit stronger cytokine responses in monocytes, while exact dosage seems less critical (47, 93–95). Additionally, an early 1980s study found significant variation between freeze dried and fresh liquid vaccines given either intradermally or IV (96). A recent Guinean trial found that BCG-Russia does not enhance innate immunity in the same way as BCG-Denmark, and BCG-Russia induces only short-lived effects on CD8+ T-cell reactivity to *C. albicans* (97). BCG-Russia is among the most widely used vaccine strains worldwide (98) but few studies used it within this review, and further research into BCG-Russia and its descendant strains is warranted. Not all included studies were methodologically clear about the BCG strain used or the preparation or immunisation methods, which should be addressed going forward, especially considering pertinent recent developments in new routes of delivery such as aerosolised BCG (99). Finally, a small trial of inactivated gamma-irradiated BCG vaccine found that it did not confer protective heterologous immunity to endotoxins, suggesting that inactivated alternatives to BCG are unlikely to deliver the same benefits as live strains (100).

#### 3.3.3 Exogenous factors

Local environmental factors may confound the outcomes measured. Although broadly-speaking the protection BCG confers against TB increases with distance from the equator, heterologous effects appear to stratify in the opposite direction, possibly through priming from maternal vaccination in neonates or enhanced immunity resulting from continual exposure to circulating mycobacteria (25). Other research suggests that BCG might influence how the immune system responds to different neonatal vaccines (101). It is possible that the opposite effect occurs, whereby variations in individual vaccine status and the surrounding pathogenic environment result in different heterologous outcomes (91, 102, 103).

Several individual factors are posited as causes of variation, such as age at time of vaccination and sampling – the studies in this review often sampled either exclusively infants or adults. BCG vaccination RCTs in the elderly indicate that both innate immunity and adaptive mechanisms contribute to heterologous effects, especially against respiratory viruses (104, 105). However, neonatal cells respond to BCG in a fundamentally distinct way to those of adults and demonstrate shifting cytokine and epigenetic profiles throughout development (9, 91, 106, 107). Immune ontogeny is therefore likely a strong factor causing variability in heterologous immunity (108).

Research also indicates that differential levels of circulating Vitamin A metabolites down-regulate trained immunity epigenetically (109), but increased release of muramyl dipeptide, a potent adjuvant, is conversely associated with a strengthened inflammatory response (110). Finally, increasing evidence indicates that individual circadian rhythms and gut microbiota such as *Roseburia* may impact heterologous effects (111–113). For example, early morning vaccination produces superior cytokine production upon heterologous stimulation compared to evening vaccination (114). There is a need to better measure, control and stratify potential exogenous and endogenous confounders to fully validate heterologous mechanisms *in vivo*.

## 4 Discussion

This review is the first to systematically synthesise literature pertaining specifically to the immune mechanisms mediating the heterologous effects of the BCG vaccine and has identified several pathways that are likely non-mutually exclusive. It is now clear that BCG vaccination induces ‘training’ of innate cells including monocytes, NK cells, and neutrophils (and their precursors) via a complex interplay of metabolic and epigenetic changes conferring immunity for a year or longer. BCG may also induce heterologous reactivity of T and B cells that is not well understood. The quality of the studies included was generally ‘good’ or ‘very good’, although many were limited in their sample size. Some of the more robust preclinical studies or clinical trials of the heterologous effects of BCG vaccination were excluded as they failed to investigate the immune mechanisms involved. Those that did so almost exclusively focused on one mechanism (in the majority of cases this was trained innate immunity). A priority going forward should thus be a more comprehensive immunological analysis of samples from large *in vivo* studies of BCG; particularly using novel systems approaches to better understand the integration of different immune pathways.

Of the studies included herein, trained immunity in innate cells has been studied in the greatest depth and outcomes are, at least *in vitro*, most consistent. Recent research has also elucidated modulation of upstream pathways, focusing on hematopoietic stem and progenitor cells (115). In contrast, there is a paucity of research on the relevance of adaptive mechanisms, particularly humoral responses; much is decades old with inconsistency in results. It is important to note that different immune mechanisms (or relative contributions thereof) may underlie the heterologous effects observed against different pathogens, and in some cases these may be regulatory (73). Immune parameters mediating protection vary by pathogen life history and infection stage, and pathogens differ in their ability to evade the protective effects of heterologous immunity through strategies such as intracellular survival, antigenic variation, and immune modulation – which may also manifest differently in *in vitro* vs. *in vivo* settings. While cross-reactivity of adaptive cells is plausible for some pathogens that share molecular patterns or antigenic similarities with mycobacteria, it is a relatively rare phenomenon, and BCG epitopes associated with such protection are yet to be described (118). Trained innate immunity may be a more broadly applicable mechanism, yet also appears not to be universal.

Follow-up studies should make use of more recent technologies to gain a clearer understanding of the role of adaptive immunity (if any) in the heterologous effects of BCG vaccination, and the mechanisms by which this is mediated, including antigen identification. If, as suggested, *M.tb* infection can enhance heterologous antibody titres (84, 116), then it is of interest why even large doses of BCG, only attenuated by about ~9.5kb of DNA (117), appear unable to do so. Ideally such studies should be performed *in vivo* to avoid the pitfalls of the artificial *in vitro* environment. However, as discussed, a number of factors may influence the heterologous effects of BCG vaccination *in vivo* including the microbiome, circadian rhythms, BCG strain/formulation used, and age or sex of the individual. While difficult to implement in real-life settings, future research should aim to minimise variation and control for confounders to better delineate mediators and ensure comparability between studies. Advances in *ex vivo* immune organoid technology may provide an opportunity to better model the complexity of immunological outcomes following vaccination in a more controlled environment (119).

This review has several strengths and limitations. Using inclusive search terms, no limit on year of publication, and systematic methods increased the likelihood of being comprehensive and unbiased, although several older articles of interest were ultimately inaccessible for abstract or full text review. This review was also limited by excluding articles concerning the effects of BCG on cancer or other immunisations. Although of interest for understanding mechanisms of heterologous immunity, the interactions of BCG with neoplastic cells or attenuated pathogens (or their antigens) may be different to the induction of trained immunity towards secondary live infections. Future research on this topic may be relevant to understanding heterologous immunity to whole live pathogens. *In silico* studies (albeit small in number) were excluded to ensure the review was focussed on empirical evidence derived from experimental studies involving biological systems and to avoid the introduction of heterogeneity/bias associated with combining data across fundamentally different methodologies. However, *in silico* studies may become increasingly significant in future research with advances in artificial intelligence. Additionally, pre-prints and non-English articles were omitted, possibly overlooking contributions from underrepresented authors or regions.

The field is evolving rapidly, prompting us to re-execute the search for relevant records published over the 18 months since the initial search, identifying 15 new eligible papers that reinforce existing hypotheses and suggest new research directions. *In vitro* models of the heterologous effects of BCG are being further optimised (120), and new translational models have been proposed including pigs, although findings in this species did not corroborate the innate immunological responsiveness to BCG seen in humans and may require further optimisation (121). Notably, the role of enhanced lung immunity in mediating innate protection against heterologous respiratory bacterial infections has been highlighted, with a key role for enhanced neutrophilia that appears to be independent of centrally trained circulating monocytes (122). Additional studies have reported extensive reprogramming of lung immune cells (123), and a biphasic innate response with robust antigen-specific Th1 cell responses in the lungs following IV BCG vaccination in hamsters and mice respectively (124). Interestingly, the latter study noted a central role for CD4+ T cell feedback on tissue myeloid and epithelial cells to imprint prolonged and broad innate antiviral resistance (124). While the role of humoral immunity remains neglected, a recent NHP study found that BCG vaccination failed to enhance antibody titres against a range of heterologous pathogen antigens (116), consistent with the findings of Kimuda et al. (84).

Investigations of exogenous factors have expanded to consider how seasons influence trained immunity. Kilic et al. found that BCG vaccination during winter induced a stronger increase in pro-inflammatory cytokine production by PBMCs and NK cells following stimulation with heterologous pathogen stimuli three months later, compared to BCG vaccination in spring. Although BCG had minimal impact on the monocyte transcriptome, vaccination resulted in notable season-dependent epigenetic alterations in both monocytes and NK cells (125). The researchers suggest that BCG vaccination in winter may enhance trained immunity due to the activation and reprogramming of immune cells, especially NK cells (125). Other cell types besides monocytes, macrophages, and NK cells can contribute to trained immunity and the heterologous effects of BCG. Research shows that γδ T cells exhibit innate memory in response to BCG vaccination in healthy volunteers when stimulated by heterologous bacterial and fungal stimuli (126). Samuel et al. have also reported evidence of innate training in bovine γδ T cells following subcutaneous BCG administration and subsequent *in vitro* stimulation with *E. coli* LPS and PAM3CSK4 (127).

Specht et al. found that the genetic background of hosts affects BCG-induced antibodies that cross-react with the SARS-CoV-2 spike protein in mice (128). A more sophisticated multi-omics analysis of over 300 healthy individuals identified genetic and epigenetic predictors of baseline immunity and immune response, finding that BCG vaccination enhanced the innate immune response more-so in individuals with a dormant immune state at baseline. The authors note that epigenetic cell states function as an ‘endophenotype’ integrating signals from genotype and environment, linking them to personal immune profiles (129). A further multi-omics analysis showed that linoleic acid metabolism was correlated with the trained immunity-inducing capacity of different BCG strains, and could act as an adjuvant to enhance BCG-induced trained immunity (130). However, despite stimulation of granulopoiesis, administration of another potential adjuvant, the nitrogen-containing bisphosphonate alendronate, alongside BCG, was associated with reduced cytokine production by PBMCs against heterologous stimuli one month later in healthy individuals (131).

Recent technologies have created opportunities to enhance our understanding of monocyte functions, interactions, and gene regulation in an *in vivo* setting, which could be vital for comprehending clinical conditions. In a single cell transcriptional analysis of the host immune response to *in vivo* trained immunity by BCG, monocytes and CD8+ T cells showed heterologous transcriptional responses to LPS, with active crosstalk between the two cell types. IFN-γ was shown to play an important role in amplifying the trained immunity response, and STAT1 found to be one of the important transcription factors for trained immunity in all identified monocyte subpopulations (132). Finally, and significantly, it has been shown that BCG alters both the epigenetics and chromatic accessibility of hematopoietic stem and progenitor cells, and these effects are directly correlated with enhanced IL-1β secretion by descendant paired PBMCs following stimulation with *C. albicans* – strong evidence to support the concept that upstream progenitor modulation is implicated in the development of long-term trained innate immunity (115).

Kandasamy et al. (8), in a review of vaccine heterology, argue that a central challenge to understanding heterologous effects is that conventional immunological measurements produce limited mechanistic insight into the association between vaccination and immune outcomes observed epidemiologically. The mechanisms by which BCG protects against TB itself are still being uncovered (133) and future research requires improved models of vaccine-mediated immunity, such as controlled human or non-human primate infection models (2, 134). However, the innovative studies discussed also point to further discovery possibilities in designs and tools already available, such as metabolomic and epigenetic profiling techniques (43).

### 4.1 Conclusion

This systematic review synthesises current literature on the immune mechanisms mediating the heterologous effects of BCG vaccination. It reveals a deep and sometimes obscure history of experimental research on the impact of BCG on heterologous immunity, extending over eight decades. It finds the greatest quantity and quality of evidence for mechanisms of trained innate immunity, particularly in monocytes, NK cells and neutrophils, while the role of cross-reactive adaptive responses (in particular humoral immunity) is less clear and less well-studied.

It is striking that the BCG vaccine, painstakingly developed under the shadow of World War I (135), remains the only vaccine against the world’s leading cause of infectious disease mortality (136). Remarkably, the mechanisms of BCG-mediated protection remain unclear and defy traditional divides of innate versus adaptive immunity and the belief that immune memory is limited to the adaptive arm. Further research into this seemingly humble vaccine is therefore highly warranted.

## Data Availability

The original contributions presented in the study are included in the article/**Supplementary Material**. Further inquiries can be directed to the corresponding author.
