# Supplementary material for: Immune mechanisms mediating the heterologous effects of BCG vaccination: a systematic review
Source: Front Immunol. 2025 May 19;16:1567111. doi: 10.3389/fimmu.2025.1567111 (PMC12127298; doi:10.3389/fimmu.2025.1567111)
Supplement: Supplementary file 1 [file DataSheet1.docx]

**Supplementary information**

**
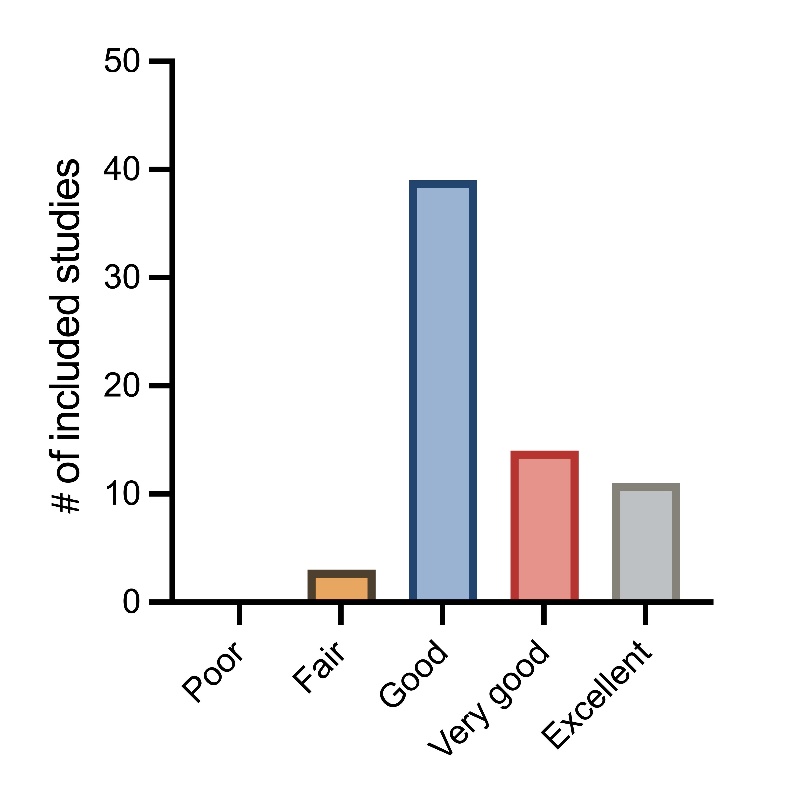
**

**Supplementary Figure 1.** Distribution of quality assessment scores for included studies.
