# Supplementary material for: Immune mechanisms mediating the heterologous effects of BCG vaccination: a systematic review
Source: Front Immunol. 2025 May 19;16:1567111. doi: 10.3389/fimmu.2025.1567111 (PMC12127298; doi:10.3389/fimmu.2025.1567111)
Supplement: Supplementary file 3 [file Table2.docx]

**Supplementary information**

| **Database** | **Search terms** |
| --- | --- |
| PubMed | ("BCG Vaccine"[MeSH Terms] OR "BCG"[Title/Abstract] OR "bacille calmette guerin"[Title/Abstract] OR "bacillus calmette guerin"[Title/Abstract] OR "tuberculosis vaccin*"[Title/Abstract] OR "tb vaccin*"[Title/Abstract] OR "calmette vaccin*"[Title/Abstract] OR "calmette guerin bacillus vaccin*"[Title/Abstract]) AND ("non-target"[Text Word] OR "off-target"[Text Word] OR "unrelated"[Text Word] OR "heterologous"[Text Word] OR "nonspecific"[Text Word] OR "non-specific"[Text Word] OR "NSE"[Text Word]) AND (("train*"[All Fields] AND "Immunity"[Text Word]) OR “innate training”[Text Word] OR "immunity, heterologous"[MeSH Terms] OR "heterologous immunity"[Text Word] OR "innate memory"[Text Word] OR "cross reactiv*"[Text Word] OR "adaptive immunity"[Text Word] OR "adaptive immunity"[MeSH Terms] OR "innate immunity"[Text Word] OR "immune memory"[Text Word] OR "cross protection"[Text Word] OR "immunity, innate"[MeSH Terms] OR "resistance"[Text Word] OR "immune protection"[Text Word] OR "epigenetic"[Text Word] OR "Th1"[Text Word] OR "polyclonal activation"[Text Word] OR "B cells"[Text Word] OR "T cells"[Text Word] OR "bystander"[Text Word]) AND "english"[Language] |
| Scopus | (INDEXTERMS("BCG Vaccine") OR TITLE-ABS(BCG) OR TITLE-ABS("bacille calmette guerin") OR TITLE-ABS("bacillus calmette guerin") OR TITLE-ABS("tuberculosis vaccin*") OR TITLE-ABS("tb vaccin*") OR TITLE-ABS("calmette vaccin*") OR TITLE-ABS("calmette guerin bacillus vaccin*")) AND (TITLE-ABS-KEY(non-target) OR TITLE-ABS-KEY(off-target) OR TITLE-ABS-KEY(unrelated) OR TITLE-ABS-KEY(heterologous) OR TITLE-ABS-KEY(nonspecific) OR TITLE-ABS-KEY(non-specific) OR TITLE-ABS-KEY(NSE)) AND ((ALL(train*) AND TITLE-ABS-KEY(Immunity)) OR TITLE-ABS-KEY("innate training") OR INDEXTERMS("immunity, heterologous") OR TITLE-ABS-KEY("heterologous immunity") OR TITLE-ABS-KEY("innate memory") OR TITLE-ABS-KEY("cross reactiv*") OR TITLE-ABS-KEY("adaptive immunity") OR INDEXTERMS("adaptive immunity") OR TITLE-ABS-KEY("innate immunity") OR TITLE-ABS-KEY("immune memory") OR TITLE-ABS-KEY("cross protection") OR INDEXTERMS("immunity, innate") OR TITLE-ABS-KEY(resistance) OR TITLE-ABS-KEY("immune protection") OR TITLE-ABS-KEY(epigenetic) OR TITLE-ABS-KEY(Th1) OR TITLE-ABS-KEY("polyclonal activation") OR TITLE-ABS-KEY("B cells") OR TITLE-ABS-KEY("T cells") OR TITLE-ABS-KEY(bystander)) AND LANGUAGE(english) |

**Supplementary Table 2. Search terms used for systematic review.**
