# Supplementary material for: Immune mechanisms mediating the heterologous effects of BCG vaccination: a systematic review
Source: Front Immunol. 2025 May 19;16:1567111. doi: 10.3389/fimmu.2025.1567111 (PMC12127298; doi:10.3389/fimmu.2025.1567111)
Supplement: Supplementary file 4 [file Table3.docx]

**Supplementary information**

| **TITLE** | **Author** | **Reference #** | **YEAR** | **SPECIES** | **REGION** | **COHORT / AGE** | **SAMPLE** | **BCG STRAIN/ ROUTE OF ADMIN** | **OUTCOMES** | **IMMUNE PARAMETERS** | **ASSOCIATIONS** |
| --- | --- | --- | --- | --- | --- | --- | --- | --- | --- | --- | --- |
| The ability of post-endotoxin serum from BCG-infected mice to induce nonspecific resistance and stimulation of granulopoiesis | Urbaschek et al. | [61] | 1982 | Vivo | Germany | NMRI, C3H/HeJ andC3HeB/FeJ, mice | NMRI= not specified. C3H/HeJ andC3HeB/FeJ 10 per group, 4 groups each mice type | M. Bovis 1029 Phipps, Lot A-17; Trudeau Institute, Saranac,N.Y/ i.v. | Effects of serum injected in mice (C3H/Hej Andc3Heb/Fej) from BCG infected mice (nmri) obtained 2 hours after injection with endotoxin | After serum transfusion from: resistance to irradiation and capability to induce granulopoiesis stimulation | Results indicate that passive transfer of BCG-ET serum is capable of inducing a distinct stimulation of granulopoiesis. The serum contains a factor(s) that mediates the elevation of CSF and CFUc, which may be essential for the induction of increased resistance to irradiation and bacterial infection |
| Activate: Randomised Clinical Trial of BCG Vaccination against Infection in the Elderly | Giamarellos-Bourboulis et al. | [104] | 2020 | Vivo | Greece | Elderly, mean age: 79.6 | Placebo (N = 78), BCG (N = 72) | Intervax, Bulgaria, Strain 1331/ i.d. | BCG's protection against heterologous pathogens in an elderly population | Incidence and character of infections, NFa, IL-10, IFN-Y, IL-6, H3K27Ac levels at promoter sites of il6 | enhanced cytokine responses to non-mycobacterial stimuli and epigenetic reprogramming of monocytes in BCG-vaccinated individuals point toward the induction of trained immunity, although it is likely that a combination between innate and heterologous T cell immunity is responsible for the entire clinical effect. |
| Adjuvant protection against bacterial infection in granulocytopenic mice | Buhles et al. | [59] | 1977 | Vivo | California, USA | Swiss-Webster mice | n=10 | Phipps Strain, Tmc 1029)/ I.P | The hypothesis tested was whether immunostimulation (BCG administration) prior to drug-induced granulocytopenia increases resistance to bacterial infection. This was evaluated using a murine model with Pseudomonas aeruginosa or Staphylococcus aureus in mice made granulocytopenic by cyclophosphamide. | Monocyte, granulocyte, lymphocyte counts, bacteremia counts of Staphylococcus and pseudomonas | Prior intraperitoneal immunostimulation of mice with BCG increased the 50% lethal dose in mice challenged subcutaneously with P. aeruginosa, but only CFA protected against challenge with S. aureus |
| Autophagy controls BCG-induced trained immunity and the response to intravesical BCG therapy for bladder cancer. | Buffen et al. | [40] | 2014 | Vitro | Dutch | Adults | n = 12 | Dutch | Response to borrelia burgdorferi, candida albicans | PBMC, cytokine array, real-time PCR, autophagy | autophagy is a central event modulating trained immunity induced by BCG |
| Bacille Calmette-Guérin induces NOD2-dependent nonspecific protection from reinfection via epigenetic reprogramming of monocytes. | Kleinnijenhuis et al. | [33] | 2012 | Vitro | Dutch | Adults | n = 20 | Denmark | Trained monocyte response to vitro Staphylococcusylococcus aureus, Candida albicans | Cytokine array, IFN-Y, Il1B, TNFa | monocytes can be functionally reprogrammed, or “trained,” to exhibit an enhanced and lasting phenotype after vaccination with BCG |
| Bacille Calmette-Guérin Vaccine Strain Modulates the Ontogeny of Both Mycobacterial-Specific and Heterologous T Cell Immunity to Vaccination in Infants | Kiravu et al. | [77] | 2019 | Vivo | Nigeria, South Africa | Infants | Group A: 84 from Jos, Nigeria, Group B:187 from Cape Town, South Africa | Group A: BCG-Bulgaria, Group B: (154 Vaccinated With BCG-Denmark And 33 With BCG-Russia)/ i.d. | Whether vaccine strains other than BCG-Denmark have a similar effect on t cell responses to unrelated antigens in newborn infants | Cell samples were then analyzed with the following antibodies IL-2-R-Pe, CD48-V500, IFN-Γ-Alexa Fluor-700, TNFα-Pe-Cy7, Ki67-Fitc, CD27-Pe-Cy5, Hla-Dr-Apc-Cy7, CD3-Bv650, CD4-Pe-Cy5.5, And CD45Ra-Pe-Texas Red-X. | The study found that the magnitude and functionality of CD4+ T cell responses to BCG and other antigens in early infancy are influenced by the specific BCG strain used for immunisation. BCG-Denmark was the most immunogenic, leading to early differentiation and a Th1 polyfunctional phenotype in memory CD4+ T cells. In contrast, BCG-Russia and BCG-Bulgaria resulted in a more naïve-like and mono-functional T cell response. Importantly, the strain of BCG also impacted responses to other vaccines like Tetanus and Pertussis, with BCG-Denmark inducing the strongest and most polyfunctional Th1 responses. The advanced memory maturation in response to BCG-Denmark may account for its enhanced T cell responses. |
| Bacille Calmette–Guérin vaccine reprograms human neonatal lipid metabolism in-vivo and in-vitro | Diray-Arce et al. | [54] | 2022 | Vivo, Vitro | Guinnea, USA, Gambia | Infants | n=99 | Denmark, India | Human neonatal lipid metabolism following BCG | Metabolites, cytokines, lipids | We report that BCG vaccination induces metabolic shifts in vivo and in vitro, particularly in lysolipid pathways, including lysophosphatidylcholines (LPCs) that correlate with Toll-like receptor (TLR)-agonist- and purified protein derivative (PPD, mycobacterial antigen)-induced whole-blood cytokine responses. |
| Bacillus Calmette-Guérin-induced trained immunity protects against SARS-CoV-2 challenge in K18-hACE2 mice | Zhang BZ et al. | [52] | 2022 | Vitro | Lab | Mice | n=19 | Japan | Response to secondary COVID-19 stimuli | COVID-19 viral load, HSCS, metabolites | BCG-i.v. vaccination protects against SARS-CoV-2 challenge in vivo. |
| Bacillus Calmette–Guérin vaccination at birth and in vitro cytokine responses to nonspecific stimulation. A randomised clinical trial | Nissen et al. | [85] | 2018 | Vivo | Dutch | Infants | n = 158 | Denmark | Response to Escherichia coli, Streptococcus, Candida | Cytokines array | No significant effects of BCG vaccination on antigen/pathogen-specific cytokine secretion |
| BCG provides short-term protection from experimental cerebral malaria in mice | Witschkowski et al. | [73] | 2020 | Vivo, In Vitro | Germany | Female C57BL/6 mice aged between 10–16 weeks | the number varied between experiments 4-8 | BCG Pasteur/S.C. For Some Experiments, BCG Was Heat Treated At 95 ◦C For 30 Min | If BCG can mediate protection from experimental cerebral malaria in mice | Surface expression of CD45, CD48, CD44, CD62L, CD160 And PD1 on leukocytes from brain, spleen and blood. Chemokine protein levels in brain homogenates. | 1. BCG Partially Protects Mice from ECM: BCG vaccination protects C57BL/6 mice from ECM (experimental cerebral malaria) induced by *Plasmodium berghei ANKA* if administered 10-30 days before infection, but not if given 70-130 days prior. Protection is linked to improved blood-brain barrier integrity.  2. Viable BCG Required for Protection: Viable BCG is necessary for protection; heat-killed BCG does not provide the same benefit.  3. No Reduction in Parasite Load: BCG does not reduce blood-stage parasitemia or parasite load in the brain, indicating protection is not due to anti-parasite effects.  4. Reduced Proinflammatory Mediators: BCG vaccination lowers proinflammatory cytokines and chemokines in the brain, leading to reduced leukocyte recruitment and inflammation, which helps prevent ECM.  5. Changes in T Cell Phenotype: BCG alters T cell responses, including reduced CXCR3+CD48+ T cells in the brain and increased PD1 expression on CD48+ T cells, suggesting BCG modulates immune responses to prevent ECM.  6. Waning Protection Over Time: Protection from ECM decreases as the time between BCG vaccination and infection increases, correlating with diminished anti-inflammatory effects and unchanged T cell responses when BCG is administered long before infection. |
| BCG Vaccination in Humans Elicits Trained Immunity via the Hematopoietic Progenitor Compartment | Cirovic et al. | [63] | 2020 | Vivo | Europe | Adults | n = 20 | Bulgaria | Induction of trained immunity | Cytokine array, CD markers, myeloid gene markers | BCG induces epigenetic, transcriptional changes in BM-derived progenitor compartments and peripheral CD14+ monocytes. Trained HSPCs can respond faster and more efficiently toward stress signals delivered, e.g., by infections. |
| BCG vaccination in humans inhibits systemic inflammation in a sex-dependent manner | Koeken et al. | [92] | 2020 | Vivo/ex Vivo | Netherlands | Adults | 303 | Bulgaria/ i.d. | Interaction between inflammation and BCG vaccination by assessing a comprehensive set of circulating inflammatory biomarkers before and after BCG vaccination | Inflammatory markers, whole blood counts of immune cell subsets. Cytokine profiles induced by M. tb and Staphylococcusylococcus aureus stimulation of PBMCs post-vaccination | We next examined if inflammatory protein profiles predicted ex vivo PBMC–derived cytokine production before and after BCG vaccination. Two weeks and 3 months after BCG vaccination, both Mycobacterium tuberculosis– and Staphylococcusylococcus aureus–induced production of innate cytokines was upregulated; Staphylococcusylococcus aureus–induced TNF-α is given as an example in Figure 8A, in both men and women (sex-specific data not shown). Females mostly showed positive correlations, whereas males only showed negative correlations between baseline circulating proteins and trained immunity responses following BCG vaccination, as for instance, clearly shown for the relationships between plasma ADA, CD5, CD48a, IL-12B, TNFRSF9, and increase in Staphylococcusylococcus aureus–induced IL-6. |
| BCG Vaccination Induces Long-Term Functional Reprogramming of Human Neutrophils | Moorlag et al. | [64] | 2020 | Vivo | Europe | Adults | n = 25 | Canada | Neutrophil response to secondary stimulation | CD16, CD10, CD11B, CD14, CD62L, Pd-L1, CD66B, CD15, and CD45, eosinophil counts | Neutrophils can be functionally reprogrammed to exhibit a propensity to respond more efficiently to microbial stimulation after vaccination with BCG and show increased activation markers and antimicrobial function ex vivo. This could be via a histone methylation pathway. |
| BCG Vaccination Protects against Experimental Viral Infection in Humans through the Induction of Cytokines Associated with Trained Immunity | Arts et al. | [42] | 2018 | Vitro | Dutch | Adults | n = 30 | Denmark | Vitro response to yellow fever vaccination | mRNA, GWAS, Cytokine Array, monocytes | Epigenetic reprogramming of monocytes induced by BCG vaccination is accompanied by significantly altered responses of innate immune cells. |
| BCG vaccination–induced emergency granulopoiesis provides rapid protection from neonatal sepsis | Brook et al. | [62] | 2020 | Vivo, Vitro | Guinnea, Lab | MICE, INFANTS | n = 26 | Denmark | Effect on granulopoiesis and activation of neutrophils | Neutrophils, TNF-A, cytokines | BCG is protective in a mouse model of neonatal polymicrobial sepsis, where it induced granulocyte colony-stimulating factor (G-CSF) within hours of administration |
| BCG-induced nonspecific effects on heterologous infectious disease in Ugandan neonates: an investigator-blind randomised controlled trial | Prentice et al. | [9] | 2021 | Vivo | Uganda | INFANTS | n = 560 | Denmark | Physician-diagnosed non-tuberculous infectious disease | Histone trimethylation at the promoter region of TNF, IL-6, And IL-1B Ex-Vivo Production Of TNF, IL-6, IL-1Β, IL-10, And IFNγ after heterologous simulation and transferrin saturation and hepcidin levels | BCG vaccination protects against non-tuberculous infectious disease during the neonatal period, in addition to having tuberculosis-specific effects |
| BCG-Induced Trained Immunity in Healthy Individuals: The Effect of Plasma Muramyl Dipeptide Concentrations | Mourits et al. | [110] | 2020 | Vivo | Europe | Adults | n = 325 | Bulgaria | Induction of trained immunity | Cytokines, HSPCs, plasma muramyl dipeptide, blood counts | Circulating MDP concentrations prior to jab are associated with the strength of trained immunity responses and influence the biological effects of BCG vaccination |
| BCG-induced trained immunity in NK cells: Role for nonspecific protection to infection. | Kleinnijenhuis et al. | [46] | 2014 | VIVIO | Dutch | Adults | n = 29 | n/r | Proinflammatory cytokine production by NK cells response to candida albicans, Staphylococcusylococcus aureus | Cytokine array, NK cells | BCG vaccination enhances the cytokine production by human NK cells after re-challenge with an unrelated microbial stimulus |
| BCG-trained innate immunity leads to fetal growth restriction by altering the immune cell profile in the mouse-developing placenta | Dang et al. | [48] | 2022 | Vivo, ex Vivo | France | Eight-week-old CBA/J (H-2k) female and DBA/2 (H-2d) male mice. Experiments were performed only on female mice either after BCG vaccination or after vaccination + mating. | 1. 6 in group of BCG. Total = 18 2. 10 in group of BCG. Total = 30 3. 7 in group of BCG. Total = 12 | Brazilian Strain; Sanofi Pasteur; 6 × 105 CFU/i.p | 1. In vivo innate immunity training of splenic and peritoneal macrophages in CBA/J female mice post BCG vaccination at day 7.  2. Effect of BCG on pregnant mice if BCG induces fetal growth restriction 3. Effect of in vivo pre-pregnancy BCG innate immune training on the maternal-fetal interface immune cell population at mid-gestation | 1. Supernatants (stimulated with LPS) of splenic and peritoneal macrophages tested for IL-10, TNF-Alpha, Or Il1-Beta. flow cytometry on splenic and peritoneal macrophages ((CD11B+F4/80+) (Markers: S (Ly6C, CD480, Icosl, Dc-Sign, CD206, CCR2).  2. Effects Of BCG on in vivo innate immune training before pregnancy on implantation, resorption rate, or gestation Length mRNA expression of some surface markers in placentas at the end of the gestation. (Markers: Klra (Expressed By NK cells), Ly6C (Expressed By Neutrophils/Monocytes/Macrophages), CD48 (Expressed By Cytotoxic T Lymphocytes), And Foxp3 (Expressed By Regulatory T-Cells)). They also, analysed the mRNA expression of several markers modified by BCG-induced trained innate immunity: CCR2 (a chemokine that specifically mediates monocyte chemotaxis) And IL-10 (an anti-inflammatory cytokine produced primarily by monocytes).  3. Flow cytometry in spleen and placentas at mid-gestation. Difference of gene expression in the developing placentas. | 1. Mice treated with BCG showed a proinflammatory profile of their splenic and peritoneal macrophages compared with those treated with PBS splenic, and peritoneal macrophages from BCG-treated mice have a significantly higher level of Ly6C. BCG training led to an increased production of proinflammatory IL1- beta by peritoneal macrophages after a proinflammatory stimulation in vitro, whereas the production of TNF-alpha was not modified. BCG vaccination did not significantly affect the expression of the surface markers on NK cells, neither in the spleen nor in the peritoneum.  2. At the end of gestation (E18.5/birth), no significant difference was detected in the number of live fetuses between the trained groups compared with the control. The resorption rate (embryonic/fetal mortality) was not significantly different between the control and trained groups. The length of the gestation was similar, approximately 20 days in all groups, suggesting BCG does not lead to preterm birth in the CBA/J x DBA/2 mouse model. However, BCG-trained groups showed a significant decrease by 12.1% of the fetal weight compared with the control (1015 ± 28 mg vs. 1154 ± 55 mg, p < 0.05)=IUGR. Placental weight was similar between groups. As the placental weight was not reduced, this suggests there is no complete growth retardation of the whole fetal–placental unit but a dysfunctional placenta that fails to assure normal fetal growth through a normal 3. Placental immune cells percentage (CD45+) were similar in the BCG-trained group and the control group, both around ∼12%. In the BCG group, the frequencies of CD11b+ myeloid cells among CD45+ cells were also similar (∼50%), with a tendency toward an increase in the BCG group. The F4/80 positive subset (macrophages) of the CD11b+ cells was reduced (1.3% vs. 11%, p = 0.0252), whereas the CD11b+ F4/80 negative subset counterpart was increased as a mirror effect. The frequency of NKp46+ CD3– NK cells amongst CD45+ immune cells were significantly lower than that of the control group (NK cells: 30% vs. 64%, p = 0.0252. For the CD3+ T cells, the frequency was slightly increased, without reaching a significant value, in the BCG group compared with the control group (6.5% vs. 2.2%, p = 0.0531. However, the level of activation of NK and T cells, assessed by CD69 expression, was not significantly different between the groups. Interestingly, in the spleen, the BCG-induced modification of Ly6C expression at the surface of macrophages was not observed during pregnancy (Figure S3(A)), but it was observed once again after pregnancy, suggesting a transient reversion of the training effect during gestation. Foxp3 gene expression did not vary between the BCG and control group, which suggests that the Treg population is not modified. Cxcl9 and Cxcl10, known to be involved in T cell chemotaxis, were not altered, but Cxcr3, known to be expressed on NK cells and involved in their recruitment to the decidua, was decreased in the BCG group, suggesting that the issue lay in a lack of effective recruitment of NK cells at the maternal-fetal interface. The BCG-trained mice also showed a significantly decreased level of Lif which is necessary for uterine blastocyst implantation in mice and is thought to play a critical role in embryo implantation in humans. As Cxcr3 is required for the recruitment of NK cells, Lif acts as a chemoattractant for macrophages,39 thus, their reduced mRNA level in E9.5 placenta could explain the significantly decreased frequency of NK cells and macrophages assessed by the flow cytometry. The mRNA level of the proinflammatory cytokines IL-6 and IFN-γ and the anti-inflammatory IL-10 were not significantly modified by BCG-induced trained innate immunity. |
| Both very low- and very high in vitro cytokine responses were associated with infant death in low-birth-weight children from Guinea Bissau | Tetteh et al. | [86] | 2014 | Vivo /Vitro | Guinea | INFANTS | n = 390 | Denmark | Infant mortality response and cytokines | Cytokine array, PPD, clinical outcomes | BCG-vaccinated children, a higher IFN-γ response to PPD was associated with better survival. Both the upper and lower extreme cytokine levels were associated with an increased mortality risk |
| CD4 T-cell-mediated heterologous immunity between mycobacteria and poxviruses | Mathurin et al. | [71] | 2009 | Vivo | Lab | ADULT MICE | n = 4 | Usa | Immune response to vaccinia virus and lymphocytic choriomeningitis virus after full antibiotic clearance | Splenomegaly, cytokines, T cell counts, viral clearance | Heterologous immunity was mediated primarily by CD4 T cells, which were shown to be activated in vivo to make IFN-γ. |
| Changes in nonspecific lymphoid (NK, K, T cell) cytotoxicity following BCG immunisation of healthy subjects | Thatcher et al. | [81] | 1978 | Vivo | England | Adults | 5 | Glaxo /Percutaneous | Nonspecific cytotoxicity after vaccination at different time points. | Lymphocytes (NK cell, K cell) | The total white blood cell count, lymphocyte count, and monocyte count showed statistically significant changes following immunisation. There was a decrease in all three cell counts on day 2 and also on day 4 for the lymphocyte count, and recovery occurred by day 7-10. The changes in lytic units for DCC, ADCC and PCC (PHA cellular Cytotoxicity), following BCG, were also statistically significant. These assays showed a decrease on day 2 (and on day 4 for ADCC), with recovery by day 7. There was an 'overshoot' on day 10 in cytotoxicity, which was still present on day 14 for DCC and on day 21 for ADCC. The increase was less evident for PCC. By day 28 all three assays had declined towards the preimmunisation values. This pattern of reactivity tended to be similar in all individuals. |
| Characterisation of the Infant Immune System and the Influence and Immunogenicity of BCG Vaccination in Infant and Adult Rhesus Macaques | Sarfas et al. | [106] | 2021 | Vivo | Lab | MACAQUES | n=78 | Denmark | Cytokine array following BCG vaccination in macaque model | Memory T cells, cytokines, B cells, granulocytes | BCG alters the frequencies of classical monocytes, CD56+ NK cells and granulocytes present in the periphery for at least three years after vaccination. |
| Circadian rhythm influences the induction of trained immunity by BCG vaccination | de Bree et al. | [114] | 2020 | Vivo | Dutch | Adults | n = 356 | Bulgaria | Induction of trained immunity | IL-6, TNF-Α, And IL-10, complete blood count, methylation markers, cytokines | BCG vaccine administration timing affects TI effect, possibly by variance in haematopoietic stem cell CLOCK genes or intrinsic neutrophil timer, or hormonal modulation therein |
| Comparison between immunopotency tests and specific active or passive acquired resistance against Mycobacterium tuberculosis in mice induced with three different preparations of BCG Pasteur vaccine | Brandely et al. | [96] | 1983 | Vivo, In Vitro | Paris, Prance | B6D2F1 (C57B1/6 X DBA/2) F1 female mice and Swiss NCS female mice - 6-8 weeks old | n/r | Pasteur/ i.v. | Activation of macrophages by theis ability to inhibit L. monocytogenes after i.v inoculation of 3 BCG preparations. Levels of nonspecific resistance were statistically higher (p < 0 001) in mice inoculated with the immuno preparation as compared to those measured in fd-treated mice. | Spleen macrophages | Nonspecific immunity is measured by macrophage's ability to inhibit L. monocytogenes after i.v BCG. Mice received 3 different preparations of BCG (Immuno, FF and FD). Nonspecific resistance was statistically higher in mice inoculated with IMMUNO. Glutamate freeze-dried vaccine =, 'Immun BCG Pasteur F', is fresh harvested preparation, distributed as an immunotherapeutic agent in a preservating medium = IMMUNo. This preparation, a well-dispersed deep culture, regularly grown in our laboratory as described previously (Lagrange & Hurtrel, 1978), was kept at - 80'C until used. In the following text, this preparation is abbreviated FF. T cell expression and macrophage activation with IMMUNO showed significantly better T cell expression and macrophage activation. |
| Correlation of increased metabolic activity, resistance to infection, enhanced phagocytosis, and inhibition of bacterial growth by macrophages from Listeria- and BCG-infected mice. | Ratzan et al. | [50] | 1976 | Vivo/In Vitro | Boston, USA | Female mice (Charles River Laboratory strain), 35 to 40 days old | Not stated | Lilly/ i.v. | Phagocytic activity and inhibition of growth of Listeria by macrophages from untreated mice and from animals injected with viable BCG, or living or killed l. monocytogenes. Macrophages were harvested from peritoneal cavities. | Phagocytosis and growth inhibition studies of macrophages incubated with Listeria | Listeria was ingested more avidly by macrophages from animals infected with either BCG or L. monocytogenes than by cells obtained from uninfected animals. The growth of Listeria was inhibited to a significantly greater degree by macrophages from BCG-infected animals than by those obtained from control mice. In the presence of normal macrophages, the multiplication of Listeria paralleled the growth of this organism in the absence of cells. Serum obtained from animals which had survived two sublethal infections with Listeria did not enhance growth inhibition. Phagocytosis of live or killed Listeria or latex particles by macrophages from BCG-infected mice was accompanied by significantly greater increases in oxidation of glucose than phagocytosis by control cells. These data indicate that macrophages from infected animals demonstrate both a higher baseline activity and greater increment in metabolism during phagocytosis than macrophages from uninfected animals. |
| Delayed BCG vaccination results in minimal alterations in T cell immunogenicity of acellular pertussis and tetanus immunisations in HIV-exposed infants | Blakney et al. | [76] | 2015 | Vivo/ex Vivo | South Africa, Cape Town | HIV exposed infants | 140 | Danish Strain 1331/Intradermal, Received At 8 Weeks Of Age | Lower TH1 responses to Staphylococcusylococcal enterotoxin b (seb) antigens in infants vaccinated at 8 weeks of age compared to those vaccinated at birth. | PBMC intracellular cytokine expression Of: Anti-CD3-Allophycocyanin (Apc)-Cyanine 7 Anti-CD48-Peridinin Chlorophyll Protein-Cy5.5, Anti-Ki67- Fluorescein Isothiocyanate (B56), Anti-IFN-Γ-Alexa Fluor 700, AntiIL-2-Apc, Anti-IL-13-Pycoerythrin, Anti-IL-17- Phycoerythrin-Cy7, And Vivid-Pacific Blue | Infants immunised with BCG at birth had higher CD4+ T cell proliferation to SEB at 14 weeks of age (p=0.018). Birth-vaccinated infants did not have increased CD48+ IL-2 expression in response to SEB, at 8 weeks. There were no observed differences in multifunctional cytokine response SEB between infants vaccinated with BCG at birth versus 8 weeks of age. There were similarly no differences at 8 weeks of age (data not shown) and of note, the majority of the proliferating T-cells were expressing none of the measured cytokines. |
| Effect of diet on nonspecific antimicrobial resistance in Mycobacterium bovis BCG-vaccinated guinea pigs | McMurray et al. | [55] | 1988 | Vivo | Lab | Guinea pigs | n = 48 | Denmark | Effect of diet on response to Listeria after BCG | Skin test reaction, number of organisms | Protein and zinc-deficient guinea pigs that did not develop skin test reactions were still resistant to listeria BCG |
| Effect of pretreatment with Bacillus Calmette-Guérin on the course of a Listeria monocytogenes infection in normal and congenitally athymic (nude) mice | Ruitenberg et al. | [70] | 1976 | Vivo | Netherlands | Male SPF BlO LP nude (nu/nu) mice | n=20 | Pasteur Institute Paris, France, Lot No. 1173-P2/ i.v. | The effect of pretreatment with BCG on the course of a listeria monocytogenes infection in athymic mice | Survival - but we kept this paper because mice are athymic. Pointing at the role of T cells in BCG-induced immunity | The data show a higher resistance after BCG treatment only in +/nu mice. Although the difference was only significant at 1 day after of L. monocytogenes challenge, this suggests that BCG pretreatment is only effective if functional T cells are present. Administration of BCG simultaneously with the Listeria challenge offered no protection. Listeria spleen counts of nude mice either treated with BCG (i.v. or i.p.) or non-treated remained virtually the same through the observation period of 35 days. This seems to indicate that BCG failed to enhance macrophage activity in mice lacking functional T cells. |
| Effects of Bacillus Calmette–Guérin (BCG) vaccination at birth on T and B lymphocyte subsets: Results from a clinical randomised trial | Birk et al. | [79] | 2017 | Vivo | Dutch | INFANTS | n = 118 | Denmark | Effect of BCG on t and b lymphocyte subset counts | CD4, CD48 T cells, CD19 B cells | No difference between the BCG group and the control group on the proportion of T or B lymphocyte subsets at 4 days post-randomisation |
| Efficacy of BCG Vaccination Against Respiratory Tract Infections in Older Adults During the Coronavirus Disease 2019 PandemiC | Moorlag et al. | [105] | 2022 | Vivo, ex Vivo | Netherlands | Adults, >60y/o | (55 placebo and 50 BCG vaccine recipients) = (Group A).  Participants without any documented SARS-CoV-2 infection (40 in the placebo and 36 in the BCG vaccine group) = Group B. 30 participants who tested positive (15 placebo and 15 BCG vaccine recipients) = Group C | Danish Strain 1331; Ssi)/ i.d. | Incidence of COVID-19 and the effect of BCG vaccination on the cellular and humoral immune responses | Group A = PBMCs Ex Vivo cytokine responses to Influenza A H1N1 California Strain And Sars-Cov-2 Wuhan Hu-1 Strain at the end of the study (Month 12). | Group A = The production of proinflammatory cytokines by peripheral blood mononuclear cells stimulated with influenza was significantly lower in the placebo than in the BCG vaccine group, with median (IQR) values as follows: interleukin 6 (IL-6), 12 550 (8396–20 237) and 17 285 (10 626–30 667) pg/mL in the placebo and BCG vaccine groups respectively (P = .04); interleukin 1β (IL-1β), 307.1 (144.9–553.8) and 467.4 (303.5–720.0) pg/mL (P = .03); and tumour necrosis factor (TNF) α, 146.5 (106.3–244.1) and 196.2 (154.7–256.8) pg/mL (P = .02). In group B the production of IL-6 was significantly lower on exposure to SARSCoV-2 in the placebo than in the BCG vaccine group (median [IQR], 1196 [782.9–1560] in the placebo vs 1646 [1109–3121] pg/mL in the BCG vaccine group; P = .048). In contrast, no difference was observed between placebo and BCG vaccine groups in the production of TNF-α, IL-1β, or interferon (IFN) γ after stimulation with SARS-CoV-2. In contrast, no difference was observed between placebo and BCG vaccine groups in the production of TNF-α, IL-1β, or interferon (IFN) γ after stimulation with SARS-CoV-2, Group C = Compared with placebo recipients, BCG vaccinated participants had significantly higher concentrations of IgG antibodies against spike protein and receptor-binding domain (Figure 3C) and IgM antibodies against spike protein |
| Enhancement of T suppressor activity in mice by high doses of BCG | Geffard et al. | [74] | 1976 | Vivo/In Vitro | France | C57B1/6, DBA/2 and (C57B1/6 x DBAz)F1 hybrids (BDFI) - 8 weeks | Not stated | Fresh Live BCG Was Kindly Provided By The Pasteur Institute Under The Form Called "Immuno BCG" Which Retains 90% Of Its Viability After 3 Months At 4 ° C | BCG can or cannot bring the formation of suppressor cells which would nonspecifically control the development of another immunologic reaction taking place later in the animal. | Level of blast transformation after exposure to phytohemagglutinin and purified protein derivative in spleen cells | Spleen cells from BCG-infected mice show impaired performance in vitro in response to both specific and nonspecific mitogens due to a suppressor T-cell population |
| Gamma-Irradiated Bacille Calmette-Guérin VaccinationDoes Not Modulate the Innate Immune Response during experimental Human Endotoxemia in Adult Males | Hamers et al. | [100] | 2015 | Vivo, In Vitro | Netherlands | Male Adults | 20 | BCG Vaccine Ssi; Statens Serum Institut, Gamma-Irradiation (25–30 Kgy)/i.d. | Effects of vaccination with gamma-irradiated BCG on the systemic innate immune response in adult males in favouring experimental endotoxemia (induced with lps from Escherichia coli o:113). LPS was administered 5 days after vaccination. | Plasma cytokines (TNF-𝛼, IL-6, IL-8,IL-10, IL-1𝛽, IL-1 Receptor Antagonist (IL-1Ra), MCP-1, AndIFN-𝛾). Analysis of leukocyte counts and differentiation. PBMCs were stimulated with mtb, heat-killed Candida albicans, Escherichia coli Lps, and Staphylococcus aureus. The same cytokines were measured in the supernatant. | BCG vaccination did not result in increased plasma levels of any of the measured cytokines in the days following vaccination. LPS administration caused transient leukocytosis in both BCG and placebo groups. Both groups went back to normal levels within 8-10 days. Five days after vaccination with BCG or placebo but before LPS administration in vivo, no differences were seen in stimulated PBMCs. In a nutshell, gamma-irradiated BCG vaccination does not influence the LPS-induced innate immune response in adult males in vivo five days later. Furthermore, no effects of BCG vaccination on the production of leukocytes stimulated ex vivo with specific and unrelated pathogens were observed |
| Glutathione Metabolism Contributes to the Induction of Trained Immunity | Ferreira et al. | [51] | 2021 | Vitro | Lab | DONOR MONO/MACROPHAGE | n/r | Canada | Metabolics of trained immunity | Glutathione, genetics, reactive oxygen species production | pharmacological modulation of glutathione and the redox status of the cell decreases the macrophage’s heterologous response, as seen by the effect of BSO or NAC on β-glucan-enhanced cytokine production |
| Hepatitis B vaccine co-administration influences the heterologous effects of neonatal BCG vaccination in a sex-differential manner | Pittet et al. | [91] | 2022 | Vivo, ex Vivo | Australia | Infants <5 with family plans to travel to high-TB incidence areas | 4-group RCT, 185 participants were randomised at birth in a 1:1:1:1 ratio to BCG, HBV, Both, or none | Danish Strain 1331 / Im | Evaluate the influence of neonatal vaccination with BCG combined with HBV on heterologous immune responses | TNF-a, IFN-y, MCP-1 in response to heterologous stimulants (Listeria, Escherichia coli, candida, Staphylococcus) | BCG-induced modulation of neonatal innate immune response results in lower cytokine responses to unrelated pathogens. HBV co-administration did not have any significant impact on responses overall but had a sex-differential effect in relation to its influence on the effect of BCG |
| Heterologous immunological effects of early BCG vaccination in low-birth-weight infants in Guinea-Bissau: a randomised-controlled trial. | Jensen et al. | [90] | 2015 | Vivo | Guinea | Infants | n = 467 | Denmark | Cytokine response to TLR agonists | Interleukin (il)-1β, IL-5, IL-6, IL-10, IL-17, interferon (IFN)-γ and tumor necrosis factor (TNF) | BCG increased the responses of Th1-polarizing cytokine (IFN-γ), increased more proinflammatory than anti-inflammatory responses (TNF-α: IL-10 ratio), and induced a higher production of cytokines typically derived from monocytes (IL-1β, IL-6, TNF-α). |
| Human Newborn Monocytes Demonstrate Distinct BCG-Induced Primary and Trained Innate Cytokine Production and Metabolic Activation In Vitro | Angelidou et al. | [108] | 2021 | Vitro | Lab | Infants | n=7 (monocyte donor) | Denmark | Metabolic pathways (chromatin remodelling) | Lactate, monocytes, cytokine array | BCG-induced human monocyte primary- and memory-innate cytokine responses were age-dependent and accompanied by distinct immunometabolic shifts that impact both glycolysis and training |
| Immunometabolic Pathways in BCG-Induced Trained Immunity | Arts et al. | [53] | 2016 | Vitro | Lab | Adults, mice | n = 12 | Denmark | Metabolic pathways (chromatin remodelling) | Glycolysis, glutamine metabolism in monocytes | β-glucan-induced trained immunity results in metabolic changes (the Warburg effect), which is the result of epigenetic modulation, e.g., increased H3K4me3 and H3K27ac at promoter sites of essential glycolytic genes, contributing to trained immunity of monocytes |
| Impaired macrophage functions as a possible basis of immune modification by microbial agents, tilorone and dimethyldioctadecylammonium bromide | Bloksma et al. | [37] | 1983 | Vivo, In Vitro | Netherlands | Swiss, BALB/c, Male mice, 12 weeks | n/r | Pasteur/ I.P | Effect on in vitro activities of macrophages, effect on nonspecific and specific resistance to Listeria monocytogenes infection | In vitro macrophage activity | 10ug BCG did not affect phagosome lysosome fusion, yeast cell phagocytosis, acridine orange uptake by lysosomes or macrophage spreading. 100ug of BCG inhibited all of the above but did not have an effect on phagosome lysosome fusion. 10/12 mice were resistant to i.p. L. monocytogenes |
| In vivo microscopic observations of the responses of Kupffer cells and the hepatic microcirculation to Mycobacterium bovis BCG alone and in combination with endotoxin | McCuskey et al. | [39] | 1983 | Vivo/In Vitro | Germany | Male NMRI (Naval Medical Research Institute) mice | n=42. Seven experimental groups were established, and each group consisted of six animals. | M. Bovis, 1029 Phipps, Lot A-17; Trudeauinstitute, Saranac, N.Y./Tale Vein | Effect that BCG infection has on mice treated with minute and toxic doses of Escherichia coli 0111 endotoxin alone and in combination | 1. kupffer cell function was assessed by measuring the rate of phagocytosis of 0.81-p.m latex particles by individual cells. 2. the relative adequacy of blood perfusion through the sinusoids was also evaluated. | In BCG-infected mice, the ratio of Kupffer cells which phagocytosed latex to sinusoids containing blood flow and capable of delivering these particulates to Kupffer cells was significantly greater than that in uninfected mice. The phagocytosis of single latex particles by individual Kupffer cells was also more rapid. This indicated an expansion of the numbers and activation of Kupffer cells. In this hyperreactive state, the tolerance-inducing dose of endotoxin produced no change in the rate of phagocytosis after 2 h. In contrast, the 70% lethal dose reduced the rate by 123%, unless tolerance was induced, in which case there was no reduction in the rate of phagocytosis. Twenty-four hours after injection of the tolerance-inducing dose, however, the rate of phagocytosis was accelerated slightly (17%). This suggested that the Kupffer cells had been activated and perhaps were more effective in clearing subsequent endotoxin from the blood but without sufficient release of toxic substances to be lethal. |
| Involvement of inflammatory cytokines and nitric oxide in the expression of nonspecific resistance to Listeria monocytogenes in mice induced by viable but not killed Mycobacterium bovis BCG | Yang et al. | [94] | 1997 | Vivo | Lab | Adult mice | n/r | Pasteur | Resistance to Listeria monocytogenes | Cytokine array, mRNA | Nitric oxide is an important mediator for the nonspecific anti-listerial activity induced by viable BCG, and IFN-c, IL-1a and TNF-a may play a critical role in the nonspecific antilisterial activity |
| Long-lasting effects of BCG vaccination on both heterologous Th1/Th17 responses and innate trained immunity | Kleinnijenhuis et al. | [80] | 2014 | Vivo | Dutch | Adults | n = 18 | n/r | Immune response to Candida albicans, Staphylococcusylococcus aureus, Escherichia coli | Cytokine, CD4 and CD48, helper T cells | BCG vaccination induces nonspecific potentiation effects of both innate trained immunity and heterologous T helper responses that are clearly present for at least 1 year after vaccination. |
| Macrophages in resistance to rickettsial infections: protection against lethal Rickettsia tsutsugamushi infections by treatment of mice with macrophage-activating agents | Nacy et al. | [36] | 1984 | Vivo/In Vitro | Washington, USA | Male, 6-to 8-week-old BALB/cl (resistant to rickettsiae) and C3H/HeJ (susceptible to rickettsiae) mice | 40 mice of each type. Total 80 | Phipps Substrain 1029/ Intradermal | If macrophages from BCG-vaccinated mice can destroy intracellular rickettsiae (they performed rickettsicidal on peritoneal cells) | Microbicidal activity of peritoneal macrophages | Resident peritoneal macrophages obtained from BALB/c mice inoculated with BCG were microbicidal for Orientia tsutsugamushi |
| Minimal Sex-Differential Modulation of Reactivity to Pathogens and Toll-Like Receptor Ligands following Infant Bacillus Calmette–Guérin Russia Vaccination | Darboe et al. | [97] | 2017 | Vitro | Gambia | Infants | n = 126 | Russia | Reactivity to innate ligands and a panel of common pathogens (Escherichia coli, Listeria, Streptococcus) | Cytokines, antibody data | No evidence that BCG vaccination of 6-week-old Gambian infants alters innate immunity and have shown that Th1 and IL-17 cytokine responses were more likely to increase over time in the BCG naïve individuals. However, the BCG naïve group also had decreased pro- to anti-inflammatory cytokine ratios (IFN-γ:IL-4, IFN-γ:IL-10, and TNF-α:IL-10) and increased IL-10 to certain stimuli, indicating a bias toward an anti-inflammatory response |
| Molecular analysis of nonspecific protection against murine malaria induced by BCG vaccination | Parra et al. | [72] | 2013 | Vivo | Lab | Adult mice | n = 10 | Pasteur | Response to intraperitoneal challenge with murine malaria | Gene expression, parasitemia | BCG immunisation induces the expression of at least 18 genes, including the antimicrobial molecules lactoferrin, eosinophil peroxidase, eosinophil major basic protein and the cathelicidin-related antimicrobial peptide (CRAMP) |
| Monocytes from neonates and adults have a similar capacity to adapt their cytokine production after previous exposure to BCG and β-glucan | Namakula et al. | [45] | 2020 | Vitro | Europe | Adults, infants | n = 60 | Bulgaria | Cytokine production following BCG stimuli | IL-6, IL-10 and TNF | BCG and β-glucan induced increase in cytokine production, reminiscent of trained immunity, showed similar levels in neonatal and adult monocytes |
| Mycobacterial infection primes T cells and macrophages for enhanced recruitment of neutrophils | Appelberg et al. | [60] | 1992 | Vitro | Lab | Adult mice | n/r | Pasteur | Recruitment of neutrophils in response to endotoxin | Peritoneal neutrophil accumulation | T cells and macrophages are primed to attract neutrophils in vivo |
| Mycobacterium tuberculosis infection is associated with increased B cell responses to unrelated pathogens | Kimuda et al. | [84] | 2020 | Vivo | Uganda | Humans, Age range: uninfected: 1-66. LTBI: 1-66, APTB: 18-53 | 68 uninfected controls, 62 individuals with latent TB infection (LTBI) and 107 active pulmonary TB (APTB) cases, and 24 recently BCG-vaccinated adolescents and naïve controls. | n/r | Effects of m.tb infection and BCG vaccination on b cell responses to heterologous pathogen recall antigens. | Antibody responses to heterologous pathogen recall antigens in study groups.  Polyclonal activation of memory bcs (mbcs) was as a possible mechanism by studying frequencies of tetanus toxoid (tt)-specific plasmablasts and mbcs in the active pulmonary TB cases and healthy donors. | BCG vaccination may not be able to boost antibody responses to heterologous pathogens, or at least not within the 3-week time frame. (Antigens: PPD purified protein derivative, TT tetanus toxoid, DT diphtheria toxoid, RSV respiratory syncytial virus, MV measles virus, CMV cytomegalovirus, EBV Epstein-Barr virus.) |
| Neonatal BCG Vaccination Influences Cytokine Responses to Toll-like Receptor Ligands and Heterologous Antigens | Freyne et al. | [88] | 2018 | Vivo | Australia | Infants | n = 212 | Denmark | Response to stimulant Staphylococcusylococcus, Streptococcus, Candida, LPS, TLR agonists | Cytokines array | BCG-vaccinated infants had increased production of IL-6 in unstimulated samples and decreased production of interleukin 1 receptor antagonist, IL-6, and IL-10 and the chemokines macrophage inflammatory protein 1α (MIP-1α), MIP-1β, and monocyte chemoattractant protein 1 (MCP-1) following stimulation |
| Neonatal BCG vaccination is associated with a long-term DNA methylation signature in circulating monocytes | Bannister et al. | [43] | 2022 | Vivo | Australia | Infants | n=130 | Denmark | Monocyte methylation | PBMC, cytokine array, gene expression, DNA methylation | Our findings indicate that the off-target effects of BCG in infants are accompanied by epigenetic remodelling of circulating monocytes that last more than 1 year. |
| Neonatal BCG Vaccination Reduces Interferon-y Responsiveness to Heterologous Pathogens in Infants From a Randomized Controlled Trial. | Curtis, N. et a | [87] | 2020 | Vitro | Australia | Infants | n = 167 | Denmark | Cytokine production 7 months afterwards to other pathogens in vitro | Cytokines IFN-y, | Lower IFN-γ responses to heterologous stimulants and TLR ligands compared with BCG-naive infants. |
| Nitric oxide involvement in experimental Trypanosoma cruzi infection in Calomys callosus and Swiss mice. | de Oliveira et al. | [58] | 1997 | Vivo | Brazil | Swiss Mice 18-22g and Calomys callosus | 20 | Unknown / Peritoneal | Nitric oxide release during parasitic trypanosomal infection + BCG inoculation | Nitric oxide, H2O2 release, parasitemia | The intraperitoneal inoculation of BCG did not induce Nitric oxide production in C. callosus during Trypanosoma cruzi infection |
| Off-target effects of bacillus Calmette–Guérin vaccination on immune responses to SARS-CoV-2: implications for protection against severe COVID-19 | Messina et al. | [78] | 2022 | Vivo | Australia | Adults | n=67 | Denmark | SARS-CoV-2 reaction | Cytokine array, effector memory T cells | The immunomodulatory signature of BCG’s off‐target effects on SARS‐CoV‐2 is consistent with a protective immune response against severe COVID‐19. |
| Plasma metabolome predicts trained immunity responses after antituberculosis BCG vaccination | Koeken et al. | [57] | 2022 | Vivo | Europe | Adults | n=325 | Bulgaria | Plasma metabolites after BCG | PBMC, secondary assay for CD3, TCR | Circulating metabolites at baseline were able to predict trained immunity responses at 3 months after vaccination and enrichment analysis based on the metabolites positively associated with trained immunity revealed enrichment of the tricarboxylic acid |
| Role of macrophages in resistance of mice to experimental cryptococcosis | Monga D.P. et al. | [38] | 1981 | Vivo, In Vitro | India | Swiss, albino mice, 6-8 weeks | n=20 | Not Stated/ i.v. | Effect of macrophage stimulation by i.v BCG on the course of cryptococcal infection | Macrophages | The mice whose macrophages were stimulated as a result of BCG treatment showed marked resistance to a massive challenge of 100 LD50 of C. neoformans. These BCG-treated animals showed only 40% mortality during a period of 60 days of observation, whereas all of the animals in the control group died during the first 10 days of the observation period. |
| Shared antigens between heterologous bacterial species | Minden et al. | [83] | 1972 | Vivo/In Vitro | Colorado, USA | Rabbits | 30 healthy, 30 immunised | Suspension Of Killed, Washed, Sonically Treated M. Bovis Strain BCG Was Obtained From The U.S.-Japan Medical Science Program/ I.M | To investigate the extent to which antibodies raised after BCG will react with antigens derived from unrelated organisms | Antibody binding to heterogeneous antigens | Antibodies in sera from normal rabbits were heterogeneous with respect to their capacity to bind with nine radiolabeled test antigens derived from taxonomically unrelated organisms (M. bovis BCG, L. monocytogenes, B. abortus, Escherichia coli, S. Typhimurium, S. epidermidis, Pseumodomas sp, H. influenzae, Corynebacterium sp). In every way, except in degree, antibodies in normal sera appeared to have the same immunological characteristics as did antibodies in sera from immunised animals. The widespread cross-reactivity between microorganisms suggests that immunological responses to bacteria may sometimes represent an elevation of an immune state rather than a new, actively acquired immune response. Resistance to infectious diseases may, in part, depend upon a preexisting state of sensitisation to bacteria that are ubiquitous in the environment. |
| Single-cell transcriptomic profiles reveal changes associated with BCG-induced trained immunity and protective effects in circulating monocytes | Kong et al. | [41] | 2021 | Vitro | Lab | Adults | n=3 (monocyte donors) | Bulgaria | Induction of trained immunity | Gene expression | The transcriptional response of monocytes after BCG vaccination, |
| Studies on the mechanism of nonspecific resistance to Brucella induced in mice by vaccination with BCG | Sulitzeanu et al. | [34] | 1961 | Vivo/In Vitro | Jerusalem, Israel | Random-bred white mice of both sexes | n=15 | Living Danish Strain. Heat-Killed = Heating The Suspension At 70 C For 60 Minutes / Intra Abdominally. | Mechanism of the nonspecific resistance to Brucella induced in mice by BCG. | The peritoneal macrophages, the spleen, the liver and the blood serum | Three defence mechanisms were examined in BCG-injected mice for their role in Brucella resistance: peritoneal macrophages, spleen and liver, and blood serum. Enhanced phagocytosis in the peritoneal cavity led to increased bacteria in mesenteric lymph nodes and reduced bacteria in the spleen and liver. BCG-treated mice showed rapid macrophage mobilisation and significant bacterial destruction in the spleen and liver. However, serum factors' role in resistance was unclear. Despite effective passive protection, serum factor presence didn't correlate with infection resistance due to experimental variability. |
| Suppressor cells induced by BCG release nonspecific factors in vitro which inhibit DNA synthesis and interleukin-2 production | Colizzi et al. | [75] | 1983 | Vivo | Italy, Uk | CBA mice | n=10 | BCG Glaxo, Middlesex, UK/i.v. or i.d. | Whether reduced hypersensitivity reactions and IL-2 production in mice are due to a suppression of DNA synthesis after BCG induction | IL-2 production, CON A induced DNA synthesis | T cells and macrophages from mice infected intravenously with high doses of BCG produce soluble factors in vitro, which inhibit Con A-induced DNA synthesis. These cells, which release nonspecific inhibitors, occur in the spleen of BCG-infected mice at the time when the animals are anergic and lack skin reactions to PPD. |
| The effect of BCG on iron metabolism in the early neonatal period: A controlled trial in Gambian neonates | Prentice et al. | [55] | 2015 | Vivo | Gambia | Infants | n = 120 | Denmark | Iron status in the first 5-days (to determine hepcidin-mediated hypoferraemia) | Iron levels, hepcidin, haemoglobin, cytokines | No evidence that BCG or other routine immunisations at birth significantly impact iron metabolism |
| The host response to Calmette-Guérin bacillus infection in mice | Blanden et al. | [35] | 1969 | Vivo, Vitro | NY, USA | Mice (6-8 weeks old) | n=30? | Rosenthal | Host's resistance to listeria monocytogenes and salmonella typhimurium after BCG (dispersed by ultrasound) | Bactericidal activity of peritoneal macrophages | The rate of onset and magnitude of the resistance produced by BCG varied with the vaccination dose. Increased resistance was detected within 48 hr of injecting large numbers of BCG (approximately 10(8) viable units), but concurrent treatment with isoniazid interrupted its further development. An equal number of heat-killed organisms failed to influence host resistance significantly. The development of tuberculin sensitivity was also dependent upon the continued survival of the immunising population of BCG. When vaccinated mice were reinfected with BCG, host resistance in the spleen and liver was rapidly augmented to the accompaniment of striking changes in the morphology and microbicidal activity of the peritoneal macrophages. These changes occurred most rapidly in mice with a high level of delayed hypersensitivity at the time of reinfection. |
| The impact of BCG dose and revaccination on trained immunity | Debisarun et al. | [47] | 2023 | Vivo | Dutch | Adults | n=51 | Denmark | Impact of dosage on trained immunity | Cytokine array, PBMC | BCG revaccination or high-dose BCG administration do not contribute to an enhanced trained immunity program beyond that induced by a standard BCG dose. |
| The influence of the gut microbiome on BCG-induced trained immunity | Stražar et al. | [113] | 2021 | Vivo | Dutch | Adults | n=321 | Bulgaria | Induction of trained immunity | Gut genome, Roseburia, cytokine array | Roseburia is found to alter both trained immune responses and phenylalanine metabolism, revealing microbes and microbial products that may alter BCG-induced immunity. |
| The role of IL-32 in Bacillus Calmette-Guérin (BCG)-induced trained immunity in infections caused by different Leishmania spp | Silva et al. | [44] | 2021 | In Vitro, Vivo | Brazil | PBMCs from BCG naïve donors. Transgenic mice for human IL-32γ ( 6- to 8-week-old C57BL/6 wild-type (WT) and IL-32γTg mice). | N/A - PBMC cultures | BCG-Intervax, Strain Sofia (Bb-Ncipd Ltd Sofia, Bulgaria)/ i.v. For Monocyte Training: Lyophilized-BCG Strain Moreau | 1. Capacity of BCG-trained monocytes to control infection caused by Leishmania amazonensis, Leishmania braziliensis or Leishmania infantum 2. Clinical outcomes of infections with these Leishmania spp. in BCG-trained mice were evaluated in the IL-32γt mouse model. | 1. TNFα and IL-32 levels in supernatants of trained and non-trained monocytes stimulated with lps from Escherichia coli. ROS production in trained monocytes.  2. parasite load in infected footpad, spleen and liver. The capacity of bone marrow monocyte-derived macrophages to kill L. infantum | BCG’s ability to train innate immune cells, providing protection against leishmaniasis, as well as the participation of IL-32γ in this process, pave the way for new treatment strategies for this neglected infectious disease. 1. The efficacy of BCG for the induction of training was confirmed in separate experiments. L. braziliensis infected approximately 80% of macrophages after 2 h and the infection index was significantly reduced after 48 h, indicating the killing of the parasites. Training with BCG significantly decreased the percentage of macrophages infected with L. braziliensis (from 2 h to 48 h) and the infection index. Overall, the data showed that training with BCG reduced the parasite load in L. braziliensis- and L. infantum-infected macrophages, whereas it increased the parasite load in L. amazonensis. However, in the end, the infection with this parasite was also controlled. Human monocytes trained with BCG showed enhanced ROS production capacity after infection with Leishmania spp. The study evaluated ROS and NO production in BCG-trained human monocyte-derived macrophages to control Leishmania infection. Results showed that Leishmania spp. Induced low ROS levels in non-trained macrophages but significantly higher ROS levels in BCG-trained macrophages. Notably, L. braziliensis infection led to higher ROS production compared to L. amazonensis or L. infantum. No significant NO production was detected under any conditions. IL-32 production and IL-32γ mRNA expression were significantly increased in BCG-stimulated monocytes compared with controls. 2. During L. amazonensis infection, BCG did not affect lesion development in WT or IL-32γTg mice. However, BCG-trained IL-32γTg mice had significantly larger lesions than BCG-trained WT mice at the infection's end. Despite this, BCG did not change parasite loads in footpad lesions in either mouse group. BCG reduced parasite numbers in the spleen and liver of both WT and IL-32γTg mice, with no parasites in the liver of BCG-trained IL-32γTg mice, indicating that BCG training effectively prevents L. amazonensis dissemination, especially with human IL-32γ. In experimental visceral leishmaniasis caused by L. infantum, BCG's effects were studied in WT and IL-32γTg mice. BCG administration increased spleen and liver weights, indicating greater hepatosplenomegaly than L. infantum alone. BCG reduced L. infantum levels in the spleen, liver, and bone marrow of both mouse types. IL-32γ and BCG together improved parasite control in the bone marrow, with bone marrow MDM from IL-32γTg and BCG-trained WT mice showing better parasitism control than PBS-injected WT cells. After BCG training, challenging with L. infantum increased the number of mononuclear cells in the spleens of both WT and IL-32γTg mice compared to controls. IL-32γTg mice showed a reduction in spleen erythrocytes, especially after BCG injection, indicating reduced spleen congestion with IL-32γ. BCG also increased granuloma areas in both mouse types post-challenge with L. infantum. |
| Variation of growth in the production of the BCG vaccine and the association with the immune response. An observational study within a randomised trial | Biering-Sørensen et al. | [93] | 2015 | Vivo | Guinea | Infants | n = 1663 | Denmark's Slow And Fast Growth | Scar formation PPD, adverse events relating to type of strain (slow or fast growth) | Cytokines, clinical outcomes | The Slow growth batches were associated with more and larger scars and a higher frequency and magnitude of the in vivo responses to PPD. Moreover, we found indications that the growth batches induced higher cytokine responses in monocytes. |
| Vitamin A induces inhibitory histone methylation modifications and down-regulates trained immunity in human monocytes. | Arts et al. | [109] | 2015 | Vitro | Dutch Lab | Adults | n = 6 donors | Denmark | Response to Escherichia coli stimulation, particularly vitamin A metabolite | Cytokines, mRNA | Our observations support the hypothesis that BCG vaccination and VAS interact and that VAS has some role in epigenetic modification similar to BCG |
| Whole Blood Profiling of Bacillus Calmette–Guérin -Induced Trained Innate Immunity in Infants Identifies Epidermal Growth Factor, IL-6, Platelet-Derived Growth Factor-AB/BB, and Natural Killer Cell Activation | Smith et al. | [107] | 2017 | Vitro | UK | Infants | n = 39 | Denmark | Response to LPS, Candida, Staphylococcusylococcus, Streptococcus, Escherichia coli | Cytokine, chemokine panel | A biosignature of BCG-induced trained innate immunity in infants comprising increases in EGF, IL-6, and PDGF-AB/BB, all of which were apparent in responses to three different innate stimuli. This is different to the characteristic signature of trained innate immunity in adults, which includes TNFα and IL-1β as well as IL-6. |

**Supplementary Table 3. Data extracted from included studies.** ADA: Adenosine Deaminase, BCG: Bacillus Calmette–Guérin, CD4+: Cluster of Differentiation 4 Positive, CFA: Complete Freund’s Adjuvant, CFUc: Colony-Forming Unit, CMV: Cytomegalovirus, COVID-19: Corona VIrus Disease of 2019, CSF: Colony-Stimulating Factor, CXCL9: Chemokine Ligand 9, CXCR3: CXC Chemokine Receptor 3, CXCRr3: CXC Chemokine Receptor 3, DNA: Deoxyribonucleic acid, EBV: Epstein-Barr Virus, ECM: Experimental Cerebral Malaria, GWAS: Genome-wide association studies , HSPCs: Hematopoietic Stem and Progenitor Cells, i.d.: intradermal, i.v.: Intravenous, IFN-γ: Interferon Gamma, IgG: Immunoglobulin G, IL-10: Interleukin 10, IL-1β: Interleukin 1 Beta, IL-6: Interleukin 6, IL: Interleukin, IUGR: Intrauterine Growth Restriction, LIF: Leukemia Inhibitory Factor, LPCs: Lysophosphatidylcholines, Ly6C: Lymphocyte Antigen 6 Complex, MDP: Muramyl Dipeptide, mRNA: messenger Ribonucleic acid , n/r: Not reported, NK: Natural Killer (cells), PBMC: Peripheral Blood Mononuclear Cells, PPD: Purified Protein Derivative, SARS-CoV-2: Severe Acute Respiratory Syndrome Coronavirus 2, TGF: Transforming Growth Factor, TH1: T helper cell, TLR: Toll-Like Receptor, TNF-α: Tumor Necrosis Factor Alpha, TNFRSF9: Tumor Necrosis Factor Receptor Superfamily, Member 9, Tregs: Regulatory T cells.
