# Supplementary material for: Immune mechanisms mediating the heterologous effects of BCG vaccination: a systematic review
Source: Front Immunol. 2025 May 19;16:1567111. doi: 10.3389/fimmu.2025.1567111 (PMC12127298; doi:10.3389/fimmu.2025.1567111)
Supplement: Supplementary file 5 [file Table4.docx]

**Supplementary information**

| **Publication and reference number** | **Author** | **Reference #** | **Pubmed_id** | **Objective** | **Population** | **Sample size** | **Intervention** | **Randomisation** | **Controls** | **Timeframe** | **Blinding** | **Methods** |  | **Outcome measured** | **Statistical analysis** | **Quality score** | **Quality category** |
| --- | --- | --- | --- | --- | --- | --- | --- | --- | --- | --- | --- | --- | --- | --- | --- | --- | --- |
|  |  |  |  | 1. Is the study question or objective clearly stated? | 2. Are the study population(s) pre-specified and clearly described? | 3. Is the sample size sufficiently large to provide confidence in the findings? | 4. Are any interventions clearly described and delivered consistently? | 5. Where individuals are assigned to an intervention, is the method of randomisation adequate (i.e., use of randomly generated assignment)? | 6. Are appropriate control group(s) included? | 7. Is the timeframe sufficient so that one could reasonably expect to see an association between intervention and outcome if it existed? | 8. Were the people assessing the outcomes blinded to the participants' interventions? | 9. Are the assay methods described in sufficient detail to allow replication?* | 10. Are appropriate replicates used | 11. Are the outcome measures pre-specified, clearly defined, valid, reliable, and assessed consistently? | 12. Is the appropriate statistical analysis applied and clearly specified? Are the presented results based on predefined aims and/or objectives, and is all data adequately tabulated with baseline data clearly specified (if applicable)? |  | **Quality category (0-2=poor, 3-5=fair, 6-8=good, 9-10=very good, 11=excellent)** |
| The ability of post-endotoxin serum from BCG-infected mice to induce nonspecific resistance and stimulation of granulopoiesis | Urbaschek et al. | [61] | 6341239 | 1 | 1 | -1 | 1 | 0 | 1 | 1 | 0 | 1 | n/a | 1 | 1 | 7 | Good |
| Activate: Randomised Clinical Trial of BCG Vaccination against Infection in the Elderly | Giamarellos-Bourboulis et al. | [104] | 32941801 | 1 | 1 | 1 | 1 | 1 | 1 | 1 | 1 | 1 | n/a | 1 | 1 | 11 | Excellent |
| Adjuvant protection against bacterial infection in granulocytopenic mice | Buhles et al. | [59] | 328788 | 1 | 1 | 0 | 1 | 0 | 1 | 1 | 0 | 1 | n/a | 1 | 1 | 8 | Good |
| Autophagy controls BCG-induced trained immunity and the response to intravesical BCG therapy for bladder cancer. | Buffen et al. | [40] | 25356988 | 1 | 1 | -1 | 1 | 0 | 1 | 1 | 0 | 1 | n/a | 1 | 1 | 7 | Good |
| Bacille Calmette-Guérin induces NOD2-dependent nonspecific protection from reinfection via epigenetic reprogramming of monocytes. | Kleinnijenhuis et al. | [33] | 22988082 | 1 | 1 | -1 | 1 | 0 | 0 | 1 | 0 | 1 | n/a | 1 | 1 | 6 | Good |
| Bacille Calmette-Guérin Vaccine Strain Modulates the Ontogeny of Both Mycobacterial-Specific and Heterologous T Cell Immunity to Vaccination in Infants | Kiravu et al. | [77] | 31649662 | 1 | 1 | 1 | 1 | 0 | 1 | 1 | 0 | 1 | n/a | 1 | 1 | 9 | Very Good |
| Bacille Calmette–Guérin vaccine reprograms human neonatal lipid metabolism in-vivo and in-vitro | Diray-Arce et al. | [54] | 35508141 | 1 | 1 | -1 | 1 | 0 | 1 | 1 | 0 | 1 | n/a | 1 | 1 | 7 | Good |
| Bacillus Calmette-Guérin-induced trained immunity protects against SARS-CoV-2 challenge in K18-hACE2 mice | Zhang BZ et al. | [51] | 35446790 | 1 | 1 | -1 | 1 | 0 | -1 | 1 | 0 | 1 | n/a | 1 | 1 | 5 | Fair |
| Bacillus Calmette–Guérin vaccination at birth and in vitro cytokine responses to nonspecific stimulation. A randomised clinical trial | Nissen et al. | [85] | 28890996 | 1 | 1 | 1 | 1 | 1 | 1 | 1 | 1 | 1 | n/a | 1 | 1 | 11 | Excellent |
| BCG provides short-term protection from experimental cerebral malaria in mice | Witschkowski et al. | [73] | 33316929 | 1 | 1 | -1 | 1 | 0 | 1 | 1 | 0 | 1 | n/a | 1 | 1 | 7 | Good |
| BCG Vaccination in Humans Elicits Trained Immunity via the Hematopoietic Progenitor Compartment | Cirovic et al. | [63] | 32544459 | 1 | 1 | -1 | 1 | 0 | 1 | 1 | 0 | 1 | n/a | 1 | 1 | 7 | Good |
| BCG vaccination in humans inhibits systemic inflammation in a sex-dependent manner | Koeken et al. | [92] | 32692728 | 1 | 1 | 0 | 1 | 0 | 1 | 1 | 0 | 1 | n/a | 1 | 1 | 8 | Good |
| BCG Vaccination Induces Long-Term Functional Reprogramming of Human Neutrophils | Moorlag et al. | [64] | 33207187 | 1 | 1 | -1 | 1 | 0 | -1 | 1 | 0 | 1 | n/a | 1 | 1 | 5 | Fair |
| BCG Vaccination Protects against Experimental Viral Infection in Humans through the Induction of Cytokines Associated with Trained Immunity | Arts et al. | [42] | 29324233 | 1 | 1 | -1 | 1 | 0 | 1 | 1 | 0 | 1 | n/a | 1 | 1 | 7 | Good |
| BCG vaccination–induced emergency granulopoiesis provides rapid protection from neonatal sepsis | Brook et al. | [62] | 32376769 | 1 | 1 | 1 | 1 | 1 | 1 | 1 | 1 | 1 | n/a | 1 | 1 | 11 | Excellent |
| BCG-induced nonspecific effects on heterologous infectious disease in Ugandan neonates: an investigator-blind randomised controlled trial | Prentice et al. | [9] | 33609457 | 1 | 1 | 1 | 1 | 1 | 1 | 1 | 1 | 1 | n/a | 1 | 1 | 11 | Excellent |
| BCG-Induced Trained Immunity in Healthy Individuals: The Effect of Plasma Muramyl Dipeptide Concentrations | Mourits et al. | [110] | 32626788 | 1 | 1 | 1 | 1 | 0 | 0 | 1 | 0 | 1 | n/a | 1 | 1 | 8 | Good |
| BCG-induced trained immunity in NK cells: Role for nonspecific protection to infection. | Kleinnijenhuis et al. | [46] | 25451159 | 1 | 1 | -1 | 1 | 0 | 0 | 1 | 0 | 1 | n/a | 1 | 1 | 6 | Good |
| BCG-trained innate immunity leads to fetal growth restriction by altering the immune cell profile in the mouse-developing placenta | Dang et al. | [48] | 34533228 | 1 | 1 | -1 | 1 | 0 | 1 | 1 | 0 | 1 | n/a | 1 | 1 | 7 | Good |
| Both very low- and very high in vitro cytokine responses were associated with infant death in low-birth-weight children from Guinea Bissau | Tetteh et al. | [86] | 24714360 | 1 | 1 | 1 | 1 | 1 | 1 | 1 | 0 | 1 | n/a | 1 | 1 | 10 | Very Good |
| CD4 T-cell-mediated heterologous immunity between mycobacteria and poxviruses | Mathurin et al. | [71] | 19193795 | 1 | 1 | -1 | 1 | 0 | 1 | 1 | 0 | 1 | n/a | 1 | 1 | 7 | Good |
| Changes in nonspecific lymphoid (NK, K, T cell) cytotoxicity following BCG immunisation of healthy subjects | Thatcher et al. | [81] | n/a | 1 | 1 | -1 | 1 | 0 | -1 | 1 | 0 | 1 | n/a | 1 | 1 | 5 | Fair |
| Characterisation of the Infant Immune System and the Influence and Immunogenicity of BCG Vaccination in Infant and Adult Rhesus Macaques | Sarfas et al. | [106] | 34707617 | 1 | 1 | 1 | 1 | 0 | 1 | 1 | 0 | 1 | n/a | 1 | 1 | 9 | Very Good |
| Circadian rhythm influences the induction of trained immunity by BCG vaccination | de Bree et al. | [114] | 32692732 | 1 | 1 | 1 | 1 | 0 | 1 | 1 | 0 | 1 | n/a | 1 | 1 | 9 | Very Good |
| Comparison between immunopotency tests and specific active or passive acquired resistance against Mycobacterium tuberculosis in mice induced with three different preparations of BCG Pasteur vaccine | Brandely et al. | [96] | 6413106 | 1 | 1 | -1 | 1 | 0 | 1 | 1 | 0 | 1 | n/a | 1 | 1 | 7 | Good |
| Correlation of increased metabolic activity, resistance to infection, enhanced phagocytosis, and inhibition of bacterial growth by macrophages from Listeria- and BCG-infected mice. | Ratzan et al. | [53] | 4629124 | 1 | 1 | -1 | 1 | 0 | 1 | 1 | 0 | 1 | n/a | 1 | 1 | 7 | Good |
| Delayed BCG vaccination results in minimal alterations in T cell immunogenicity of acellular pertussis and tetanus immunisations in HIV-exposed infants | Blakney et al. | [76] | 26259542 | 1 | 1 | 1 | 1 | 0 | 1 | 1 | 0 | 1 | n/a | 1 | 1 | 9 | Very Good |
| Effect of diet on nonspecific antimicrobial resistance in Mycobacterium bovis BCG-vaccinated guinea pigs | McMurray et al. | [55] | n/a | 1 | 1 | 0 | 1 | 0 | 1 | 1 | 0 | 1 | n/a | 1 | 1 | 8 | Good |
| Effect of pretreatment with Bacillus Calmette-Guérin on the course of a Listeria monocytogenes infection in normal and congenitally athymic (nude) mice | Ruitenberg et al. | [70] | 821508 | 1 | 1 | 0 | 1 | 0 | 1 | 1 | 0 | 1 | n/a | 1 | 1 | 8 | Good |
| Effects of Bacillus Calmette–Guérin (BCG) vaccination at birth on T and B lymphocyte subsets: Results from a clinical randomised trial | Birk et al. | [79] | 28963455 | 1 | 1 | 1 | 1 | 1 | 1 | 1 | 1 | 1 | n/a | 1 | 1 | 11 | Excellent |
| Efficacy of BCG Vaccination Against Respiratory Tract Infections in Older Adults During the Coronavirus Disease 2019 PandemiC | Moorlag et al. | [105] | 35247264 | 1 | 1 | 1 | 1 | 1 | 1 | 1 | 1 | 1 | n/a | 1 | 1 | 11 | Excellent |
| Enhancement of T suppressor activity in mice by high doses of BCG | Geffard et al. | [74] | n/a | 1 | 1 | 0 | 1 | 0 | 1 | 1 | 0 | 1 | n/a | 1 | 1 | 8 | Good |
| Gamma-Irradiated Bacille Calmette-Guérin VaccinationDoes Not Modulate the Innate Immune Response during experimental Human Endotoxemia in Adult Males | Hamers et al. | [100] | 25883989 | 1 | 1 | -1 | 1 | 1 | 1 | 1 | 1 | 1 | n/a | 1 | 1 | 9 | Very Good |
| Glutathione Metabolism Contributes to the Induction of Trained Immunity | Ferreira et al. | [50] | 33919212 | 1 | 1 | -1 | 1 | 0 | 1 | 1 | 1 | 1 | n/a | 1 | 1 | 8 | Good |
| Hepatitis B vaccine co-administration influences the heterologous effects of neonatal BCG vaccination in a sex-differential manner | Pittet et al. | [91] | 35105495 | 1 | 1 | 1 | 1 | 1 | 1 | 1 | 1 | 1 | n/a | 1 | 1 | 11 | Excellent |
| Heterologous immunological effects of early BCG vaccination in low-birth-weight infants in Guinea-Bissau: a randomised-controlled trial. | Jensen et al. | [90] | 25210141 | 1 | 1 | 1 | 1 | 1 | 1 | 1 | 1 | 1 | n/a | 1 | 1 | 11 | Excellent |
| Human Newborn Monocytes Demonstrate Distinct BCG-Induced Primary and Trained Innate Cytokine Production and Metabolic Activation In Vitro | Angelidou et al. | [108] | 34326836 | 1 | 1 | -1 | 1 | 0 | 1 | 1 | 0 | 1 | n/a | 1 | 1 | 7 | Good |
| Immunometabolic Pathways in BCG-Induced Trained Immunity | Arts et al. | [52] | 27926861 | 1 | 1 | -1 | 1 | 0 | 1 | 1 | 0 | 1 | n/a | 1 | 1 | 7 | Good |
| Impaired macrophage functions as a possible basis of immune modification by microbial agents, tilorone and dimethyldioctadecylammonium bromide | Bloksma et al. | [37] | 6351737 | 1 | 1 | 0 | 1 | 0 | 1 | 1 | 0 | 1 | n/a | 1 | 1 | 8 | Good |
| In vivo microscopic observations of the responses of Kupffer cells and the hepatic microcirculation to Mycobacterium bovis BCG alone and in combination with endotoxin | McCuskey et al. | [39] | 6352499 | 1 | 1 | 0 | 1 | 0 | 1 | 1 | 0 | 1 | n/a | 1 | 1 | 8 | Good |
| Involvement of inflammatory cytokines and nitric oxide in the expression of nonspecific resistance to Listeria monocytogenes in mice induced by viable but not killed Mycobacterium bovis BCG | Yang et al. | [94] | 9049997 | 1 | 1 | 0 | 1 | 0 | 1 | 1 | 0 | 1 | n/a | 1 | 0 | 7 | Good |
| Long-lasting effects of BCG vaccination on both heterologous Th1/Th17 responses and innate trained immunity | Kleinnijenhuis et al. | [80] | 24192057 | 1 | 1 | -1 | 1 | 0 | 0 | 1 | 0 | 1 | n/a | 1 | 1 | 6 | Good |
| Macrophages in resistance to rickettsial infections: protection against lethal Rickettsia tsutsugamushi infections by treatment of mice with macrophage-activating agents | Nacy et al. | [36] | 6584528 | 1 | 1 | 0 | 1 | 0 | 1 | 1 | 0 | 1 | n/a | 1 | 0 | 7 | Good |
| Minimal Sex-Differential Modulation of Reactivity to Pathogens and Toll-Like Receptor Ligands following Infant Bacillus Calmette–Guérin Russia Vaccination | Darboe et al. | [97] | 28951731 | 1 | 1 | 1 | 1 | 1 | 1 | 1 | 0 | 1 | n/a | 1 | 1 | 10 | Very Good |
| Molecular analysis of nonspecific protection against murine malaria induced by BCG vaccination | Parra et al. | [72] | 23861742 | 1 | 1 | 1 | 1 | 0 | 1 | 1 | 0 | 1 | n/a | 1 | 1 | 9 | Very Good |
| Monocytes from neonates and adults have a similar capacity to adapt their cytokine production after previous exposure to BCG and β-glucan | Namakula et al. | [45] | 32084227 | 1 | 1 | -1 | 1 | 0 | 1 | 1 | 0 | 1 | n/a | 1 | 1 | 7 | Good |
| Mycobacterial infection primes T cells and macrophages for enhanced recruitment of neutrophils | Appelberg et al. | [60] | 1376352 | 1 | 1 | -1 | 1 | 0 | 1 | 1 | 0 | 1 | n/a | 1 | 1 | 7 | Good |
| Mycobacterium tuberculosis infection is associated with increased B cell responses to unrelated pathogens | Kimuda et al. | [84] | 32868810 | 1 | 1 | 1 | 1 | 0 | 1 | 1 | 0 | 1 | n/a | 1 | 1 | 9 | Very Good |
| Neonatal BCG Vaccination Influences Cytokine Responses to Toll-like Receptor Ligands and Heterologous Antigens | Freyne et al. | [88] | 29415180 | 1 | 1 | 1 | 1 | 1 | 1 | 1 | 1 | 1 | n/a | 1 | 1 | 11 | Excellent |
| Neonatal BCG vaccination is associated with a long-term DNA methylation signature in circulating monocytes | Bannister et al. | [43] | 35930640 | 1 | 1 | 1 | 1 | 0 | 1 | 1 | 0 | 1 | n/a | 1 | 1 | 9 | Very Good |
| Neonatal BCG Vaccination Reduces Interferon-y Responsiveness to Heterologous Pathogens in Infants From a Randomized Controlled Trial. | Freyne et al. | [88] | 31990350 | 1 | 1 | 1 | 1 | 1 | 1 | 1 | 1 | 1 | n/a | 1 | 1 | 11 | Excellent |
| Nitric oxide involvement in experimental Trypanosoma cruzi infection in Calomys callosus and Swiss mice. | de Oliveira et al. | [58] | 9342740 | 1 | 1 | 1 | 1 | 0 | 1 | 1 | 0 | 1 | n/a | 1 | 1 | 9 | Very Good |
| Off-target effects of bacillus Calmette–Guérin vaccination on immune responses to SARS-CoV-2: implications for protection against severe COVID-19 | Messina et al. | [78] | 35573165 | 1 | 1 | -1 | 1 | 1 | 1 | 1 | 1 | 1 | n/a | 1 | 1 | 9 | Very Good |
| Plasma metabolome predicts trained immunity responses after antituberculosis BCG vaccination | Koeken et al. | [57] | 36094960 | 1 | 1 | 1 | 1 | 0 | 1 | 1 | 0 | 1 | n/a | 1 | 1 | 9 | Very Good |
| Role of macrophages in resistance of mice to experimental cryptococcosis | Monga D.P. et al. | [38] | 6265377 | 1 | 1 | 0 | 1 | 0 | 1 | 1 | 0 | 1 | n/a | 1 | 1 | 8 | Good |
| Shared antigens between heterologous bacterial species | Minden et al. | [83] | 4344028 | 1 | 1 | 0 | 1 | 0 | 1 | 1 | 0 | 1 | n/a | 1 | 1 | 8 | Good |
| Single-cell transcriptomic profiles reveal changes associated with BCG-induced trained immunity and protective effects in circulating monocytes | Kong et al. | [41] | 34788625 | 1 | 1 | -1 | 1 | 0 | 1 | 1 | 0 | 1 | n/a | 1 | 1 | 7 | Good |
| Studies on the mechanism of nonspecific resistance to Brucella induced in mice by vaccination with BCG | Sulitzeanu et al. | [34] | 14039582 | 1 | 1 | -1 | 1 | 0 | 1 | 1 | 0 | 1 | n/a | 1 | 1 | 7 | Good |
| Suppressor cells induced by BCG release nonspecific factors in vitro which inhibit DNA synthesis and interleukin-2 production | Colizzi et al. | [75] | 6228520 | 1 | 1 | -1 | 1 | 0 | 1 | 1 | 0 | 1 | n/a | 1 | 1 | 7 | Good |
| The effect of BCG on iron metabolism in the early neonatal period: A controlled trial in Gambian neonates | Prentice et al. | [55] | 25959747 | 1 | 1 | 1 | 1 | 1 | 1 | 1 | 1 | 1 | n/a | 1 | 1 | 11 | Excellent |
| The host response to Calmette-Guérin bacillus infection in mice | Blanden et al. | [35] | 497610 | 1 | 1 | -1 | 1 | 0 | 1 | 1 | 0 | 1 | n/a | 1 | 1 | 7 | Good |
| The impact of BCG dose and revaccination on trained immunity | Debisarun et al. | [47] | 36565972 | 1 | 1 | -1 | 1 | 1 | 1 | 1 | 0 | 1 | n/a | 1 | 1 | 8 | Good |
| The influence of the gut microbiome on BCG-induced trained immunity | Stražar et al. | [113] | 34551799 | 1 | 1 | 1 | 1 | 0 | 0 | 1 | 0 | 1 | n/a | 1 | 1 | 8 | Good |
| The role of IL-32 in Bacillus Calmette-Guérin (BCG)-induced trained immunity in infections caused by different Leishmania spp | Silva et al. | [44] | 34260904 | 1 | 1 | -1 | 1 | 0 | 1 | 1 | 0 | 1 | n/a | 1 | 1 | 7 | Good |
| Variation of growth in the production of the BCG vaccine and the association with the immune response. An observational study within a randomised trial | Biering-Sørensen et al. | [93] | 25765965 | 1 | 1 | 1 | 1 | 1 | 1 | 1 | 0 | 1 | n/a | 1 | 1 | 10 | Very Good |
| Vitamin A induces inhibitory histone methylation modifications and down-regulates trained immunity in human monocytes. | Arts et al. | [109] | 25934925 | 1 | -1 | 0 | 1 | 0 | 1 | 1 | 0 | 1 | n/a | 1 | 1 | 6 | Good |
| Whole Blood Profiling of Bacillus Calmette–Guérin -Induced Trained Innate Immunity in Infants Identifies Epidermal Growth Factor, IL-6, Platelet-Derived Growth Factor-AB/BB, and Natural Killer Cell Activation | Smith et al. | [107] | 28634479 | 1 | 1 | 0 | 1 | 0 | 1 | 1 | 0 | 1 | n/a | 1 | 1 | 8 | Good |

**Supplementary Table 4. Quality assessment of included studies.**

Questions:

1. Is the study question or objective clearly stated?

2. Are the study population(s) pre-specified and clearly described?

3. Is the sample size sufficiently large to provide confidence in the findings?

4. Are any interventions clearly described and delivered consistently?

5. Where individuals are assigned to an intervention, is the method of randomisation adequate (i.e., use of randomly generated assignment)?

6. Are appropriate control group(s) included?

7. Is the timeframe sufficient so that one could reasonably expect to see an association between intervention and outcome if it existed?

8. Were the people assessing the outcomes blinded to the participants' interventions?

9. Are appropriate replicates used?

10. Are the assay methods described in sufficient detail to allow replication?

11. Are the outcome measures pre-specified, clearly defined, valid, reliable, and assessed consistently?

12. Is the appropriate statistical analysis applied and clearly specified? Are the presented results based on predefined aims and/or objectives, and is all data adequately tabulated with baseline data clearly specified (if applicable)?

Possible responses: Y, N, NR (not reported), NA (not applicable); Quality category (0-2=poor, 3-5=fair, 6-8=good, 9-10=very good, 11-12=excellent).
